# Host shift induces changes in mate choice of the seed predator *Acanthoscelides obtectus* via altered chemical signalling

**DOI:** 10.1371/journal.pone.0206144

**Published:** 2018-11-14

**Authors:** József Vuts, Christine M. Woodcock, Lisa König, Stephen J. Powers, John A. Pickett, Árpád Szentesi, Michael A. Birkett

**Affiliations:** 1 Department of Biointeractions and Crop Protection, Rothamsted Research, Harpenden, United Kingdom; 2 Karl-Franzens-University, Graz, Austria; 3 Department of Computational and Analytical Sciences, Rothamsted Research, Harpenden, United Kingdom; 4 School of Chemistry, Cardiff University, Cardiff, United Kingdom; 5 Department of Systematic Zoology and Ecology, Eötvös Loránd University, Budapest, Hungary; INRA-UPMC, FRANCE

## Abstract

The mechanisms of host shift in phytophagous insects are poorly understood. Among the many proposed processes involved, sexual selection via semiochemicals has recently been suggested. This hypothesizes that sexual communication using pheromones is modified as a result of development on a new host, and such plant-induced phenotypic divergence in mate recognition cues can lead to reproductive isolation between host lines. We tested this hypothesis on *Acanthoscelides obtectus*, an oligophagous bruchid of *Phaseolus vulgaris* beans worldwide, which also develops in acceptable non-hosts, such as chickpea (*Cicer arietinum* L.). Male sex pheromone blends of the bean, chickpea and chickpea/bean host lines during artificially induced host shifts showed different composition. Bean-reared females did not distinguish between blends, whereas chickpea and chickpea/bean females preferred the chickpea male pheromone. However, electrophysiological (EAG) responses to male odour of antennae of the three female host lines were similar, all preferring bean-reared males. Egg-laying choice tests revealed a uniform preference for bean seeds across female host lines, even after multiple generations, whereas larvae did not distinguish between bean and chickpea seeds. We conclude that the development of divergent chemical signalling systems during host shifts does not facilitate the evolution of host races in *A*. *obtectus*, because oviposition preferences remain unaffected.

## Introduction

Of the ca. 450,000 known phytophagous insect species, at least 70% are mono- and oligophagous, consuming a narrow range of species within one plant family [1,2]. Due to their making up a large proportion of the Insecta class (45%), the evolution of herbivorous insects strongly influences theories on the evolution of insect diversity [3]. Within herbivores, mono- and oligophagy is a more frequent strategy than polyphagy (ca. 70% vs 30%, respectively), which might reflect the relatively higher rates of speciation and extinction among mono- and oligophages [4]. The forces thought to drive the evolution of host plant specialisation in herbivorous insects include i) autonomous hereditary changes in the chemosensory system [5], ii) distribution of plant secondary metabolites [6], iii) interspecific competition [7], iv) escape from predation [8], or v) ecological fitting [9]. Populations of species undergoing host shift may then become reproductively separated by natural and/or sexual selection [10].

Sexual selection has been suggested as another mechanism driving host shift in phytophagous insects, proposing that host specialization can result from female mate choice being driven by certain diet-derived male pheromones, which induce egg-laying on a specific host plant [11]. The model predicts that, after a host shift, the ability to sequester host plant compounds into an attractive pheromone evolves first, accompanied by a pre-existing female preference for that plant-derived pheromone, which would drive specialization on that host plant by indirectly selecting for oviposition preference genes. Two recent examples emphasize the role of sexual selection in prezygotic isolation by chemical signalling during host range shift. Individuals of a leaf beetle, *Phaedon cochleariae* Fabr. (Coleoptera: Chrysomelidae), that develop on an alternative host plant show differences in their cuticular hydrocarbon profile compared to individuals on the ancestral host, and prefer to mate with beetles of the same host line [12]. Assortative mating has been observed among individuals of another chrysomelid, the dried bean beetle (*Acanthoscelides obtectus* Say), that were reared on the ancestral host, common bean (*Phaseolus vulgaris* L.) [13], as opposed to those reared on an acceptable non-host, chickpea (*Cicer arietinum* L.) (Fabaceae). (For a definition of an acceptable non-host, see [14].) Although chickpea-reared individuals had reduced mate discrimination characteristics, low level of mate choice isolation between the two host lines was found, in which the role of cuticular hydrocarbons as contact pheromones [15] was proposed. The authors speculate that plant-induced phenotypic divergence in mate recognition cues can lead to reproductive isolation between host lines [12,13].

*A*. *obtectus* is an oligophagous seed predator specializing on the *Ph*. *vulgaris* group [16]. Originating in the Neotropics, it has become cosmopolitan through human-mediated migrations since the domestication and distribution of beans [17], and can have several generations a year in the tropics and subtropics, multiplying both in the field and in granaries [18]. The species has served as a model for life history studies. Profound differences in life history traits, such as developmental time, body mass or lifespan, were found between two host lines developed on bean and chickpea [19]. Short-term (plastic) effects of host shift were also observed on certain physiological properties. For example, one-generation shift from bean to chickpea significantly increased the activity of phosphatase enzymes [20] and altered cuticular hydrocarbon profiles [15], reflecting immediate plastic responses to nutritional changes. Chemical communication in *A*. *obtectus* not only involves contact chemoreception [15,21], but also a male-produced volatile sex pheromone [22], highlighting the complex nature of intraspecific signalling in this species. In the present study, we tested the hypothesis that artificial host shift induces segregation in sex pheromone communication between two host lines of *A*. *obtectus*. Specifically, the following hypotheses were tested: 1) volatile chemical signalling changes plastically with host line formation; 2) such changes are maintained under breeding on acceptable non-host (chickpea) for multiple generations; and 3) altered chemical communication facilitates host shift that may eventually lead to reproductive isolation.

## Materials and methods

### Insect rearing

*Acanthoscelides obtectus* beetles were obtained from a laboratory population reared either on dry ‘Cannellini’ beans (*Ph*. *vulgaris*) (bean line) or chickpea seeds (*C*. *arietinum*) (chickpea line). A third `host line`was created by rearing beetles from chickpea on beans (chickpea/bean line). The original laboratory population was from a natural infestation on *Ph*. *vulgaris* in Hungary. Maintenance conditions were as follows: artificial lighting with a 16:8 h L:D photoperiod, a constant temperature of 20°C, and 60% RH. In order to obtain virgin insects, seeds were kept individually in wells of an Eppendorf rack and covered with a piece of transparent acetate sheet until beetle emergence, at which point the sexes were separated immediately for use in experiments.

### Dynamic headspace collection (air entrainment)

To determine the composition of pheromone blends, aeration extracts [23] were prepared from male *A*. *obtectus* of the three different host lines. Air was pumped (12VDC pump; KNF, Reiden, Switzerland) through an activated charcoal filter and Teflon tubing at 600 mL/min into a glass chamber (10 cm diam., 5 cm height), enclosing a single 6-8-day-old beetle, to provide a positive pressure of clean air. Males of this age were used because their volatile release peaks at this time (S1 Fig). A glass tube (8 cm × 0.3 cm i.d.) containing 50 mg Porapak Q adsorbent sandwiched between glass wool plugs was inserted into the air outlet. Air was drawn from the chamber through the tube under negative pressure at a flow rate of 500 mL/min., and at a constant temperature of 20°C. In this way, volatile compounds were collected on Porapak Q traps for 24 h and were then eluted from the adsorbent with 750 μL freshly distilled diethyl ether. Extracts were concentrated to 50 μL under a gentle nitrogen stream and kept at −20°C until use. Six biological replicates were prepared from male beetles developed in each host seed type.

### Analyses of male headspace extracts

The identity of peaks in the aeration extracts were confirmed by comparison of their gas chromatographic (GC) and mass spectrometric (GC-MS) properties with those of authentic standards, and by GC peak enhancement using authentic samples. GC conditions were: an Agilent 6890A GC equipped with a cool on-column injector, a flame ionization detector (FID) and a 50 m × 0.32 mm i.d. HP-1 column (J & W Scientific, Folsom, CA, USA). The oven temperature was maintained at 30°C for 1 min, then programmed to increase at 5°C/min to 150°C, then held for 0.1 min, then programmed to increase at 10°C/min to 250°C and then held for 20 min. The carrier gas was hydrogen. GC-MS conditions were: a Micromass Autospec Ultima magnetic sector mass spectrometer (Waters, Milford, MA) attached to an Agilent 6890N GC (fitted with a 50 m × 0.32 mm i.d. x 0.52 μm film thickness non-polar HP-1 column, J & W Scientific), and equipped with a cool-on-column injector. Ionization was by electron impact (70 eV, 220°C). The GC oven temperature was maintained at 30°C for 5 min and then programmed to increase at 5°C/min to 250°C, with a 70-min run time. Six compounds, representing the volatile sex pheromone constituents of male *A*. *obtectus*, were of interest: methyl (2*E*,4*Z*,7*Z*)-2,4,7-decatrienoate, methyl (2*E*,4*Z*)-2,4-decadienoate, (3*Z*,6*E*)- and (3*E*,6*E*)-α-farnesene, methyl (*E*,*R*)-2,4,5-tetradecatrienoate and octadecanal. Because the total amounts of compounds varied between sampled individuals, analysis of variance (ANOVA) was applied (completely randomised design) to the normalised peak area data, the normalisation consisting of calculating the ratios of the areas under the peaks for the 6 compounds to the total area. This normalisation accounts for some of the overall differences between beetles. A log-to-base 10 transformation was used to account for some heterogeneity of variance over the three treatments, with an adjustment of 0.001 to account for zero observations. ANOVA, providing an F-test for the overall difference between host lines, was followed by application of Fisher’s least significant difference (LSD) test (p < 0.05) for the statistical separation of means of most interest. The Genstat (2015, 18^th^ edition, VSN International Ltd, Hemel Hempstead, UK) statistical package was used for the analysis.

Authentic standards of the six constituents of the male *A*. *obtectus* pheromone were obtained according to the parenthetical references as follows: methyl (2*E*,4*Z*)-2,4-decadienoate [24], methyl (*E*,*R*)-2,4,5-tetradecatrienoate and methyl (2*E*,4*Z*,7*Z*)-2,4,7-decatrienoate [25], (3*Z*,6*E*)- and (3*E*,6*E*)-α-farnesene [26,27], octadecanal (by tetrapropylammonium perruthenate (TPAP)-mediated oxidation of octadecanol [28]).

### Behavioural assays

Two types of behaviour assay were used to compare the response of females from the three different host lines to the three synthetic chemical blends of pheromones of male beetles from these host lines or control (air). This provided a three (host lines) by four (blends + control) factorial treatment structure for assessment.

Firstly, a four-arm olfactometer [29] was used to measure female responses to synthetic blends. Glass arms (narrow part: 50 mm length × 2.5 mm diam., wide part: 90 mm length × 20 mm diam.) were attached through a 3 mm diameter hole to the end of each of the four arms (Figure A in S2 File). Prior to each experiment, all glassware was washed with Teepol (Orpington, UK) detergent, rinsed with acetone and distilled water and baked in an oven overnight at 160°C. Perspex components were washed with Teepol solution, rinsed with 80% ethanol solution and distilled water, and left to air-dry. The olfactometer was illuminated from above by diffuse uniform lighting from two 18W/35 white fluorescent light bulbs screened with red acetate. It was surrounded by black paper to remove any external visual stimuli. Test compounds were applied onto filter paper strips (ca. 2 cm^2^, Whatman, Little Chalfont, UK) in proportions and doses in such a way that the amounts released per hour were similar to those emitted by one male beetle over 1 h specific to each host line (Table 1). When only one blend was tested, three control arms were treated with 10 μL redistilled hexane, whereas in the case of two treatments, these were positioned in two opposite arms, accompanied by two control arms. After allowing the solvent to evaporate for 30 s, the strips were inserted into the glass arms. This setup ensured the robustness of the experiment by making it less likely for an insect to accidentally walk in or out of the treated region. A single virgin, 3-6-day-old female was introduced through a hole in the top of the olfactometer. (In comparison, mated females give weak positive response to the male pheromone; J. Vuts, pers. comm.). Air was drawn through the central hole by a vacuum pump and, consequently, pulled through each of the four side arms (75 mL/min/arm) and subsequently exhausted from the room. Each beetle was given 2 min to acclimatize in the olfactometer (the room temperature was 20°C and RH 60%), after which the experiment was run for 16 min. The olfactometer was rotated 90° every 4 min to control for any directional bias. The olfactometer was divided into four regions, corresponding to each of the four arms, and the time spent in each arm was recorded. The structure of the experiment was such that there were 18 assays (3 individual host lines plus 3 pairs of host lines by 3 blends) by 10 replicates each, giving 180 olfactometer runs altogether, but there were either two or three blend treatments (including control) tested in each one, giving an unbalance that precludes use of ANOVA. Instead, a linear mixed model (LMM), fitted using the method of residual maximum likelihood (REML), was applied to the data, which takes account of the design structure of olfactometer replicate runs and areas within them (as split-plots) before testing (F-tests) for the main effects and interaction between the factors of host line and blend. Three analyses were done: using data from all assays, using data only from the assays assessing blends in pairwise fashion, and using data only from the assays comparing individual blends. A square root transformation of the time spent data was used to account for some heterogeneity of variance over the treatment combinations, assessment of residuals revealing that they endorsed conformation to the assumptions of the analysis. Predicted means for statistically relevant (p < 0.05, F-test) terms from the model were output for comparison using Fisher’s LSD test (p < 0.05). Genstat was used for this analysis.

**Table 1:** Composition (ng/μL) of male blends used in olfactometer bioassays.

compound	host line		
	bean	chickpea	chickpea/bean
methyl (2*E*,4*Z*,7*Z*)-2,4,7-decatrienoate	5	0	7
methyl (2*E*,4*Z*)-2,4-decadienoate	40	80	40
(3*Z*,6*E*)-α-farnesene	43	35	48
(3*E*,6*E*)-α-farnesene	50	40	25
methyl (*E*,*R*)-2,4,5-tetradecatrienoate	390	390	770
octadecanal	5	5	5

Ten μL hexane solution was offered on filter paper.

Ten females from a population reared on chickpea for 50 generations were also tested against the bean and chickpea male blends and control, in 10 replicate olfactometer runs. Data from this assay was also analysed using a linear mixed model, albeit in this case to test the main effects and interaction between the factors of number of generations (1 or 50) and type of male blend (bean, chickpea or control).

Secondly, a still-air bioassay was developed to model natural situations in *A*. *obtectus* chemical communication that may occur in granaries where bean and chickpea seeds can be stored in close proximity (Figure A in S3 File). Ten virgin females from each of the bean and chickpea host lines were released in a Petri dish (14 cm diam. × 2 cm height). Individuals from the chickpea line were marked with a water-based white marker (Tipp-Ex Ecolutions, Societe Bic, France) by placing a small dot on the dorsal surface of the thorax. The arena was connected via 9 mm tubes (custom-made pipette tips, Sigma-Aldrich, UK) to three plastic vials (2.5 cm diam. × 9 cm height, Starlab, Muri, Switzerland). At the bottom of each of 2 of these vials, two male-equivalent amounts of one of the synthetic pheromone blends on a filter paper strip were placed, either typical of males from the bean or chickpea host line. In the third vial, an equal volume (20 μL) of hexane was added on a paper strip as control. This experiment was replicated 7 times. Environmental conditions were the same as those for the olfactometer tests. The number of beetles trapped inside each vial, as well as in the main arena, was determined after 24 h. The resulting data in the form of counts in a two (bean or chickpea host line female) by four (bean, chickpea, control or Petri dish) outcomes contingency table for each replicate were analysed by fitting a generalized linear model (GLM) assuming a Poisson distribution and using a natural log link function (ln), taking the replicates as a blocking factor. Having tested (Chi-squared test) the interaction between the two factors, the predicted counts for statistical comparison on the transformed scale and for presentation on the back-transformed scale were output. Comparisons were made using Fisher’s LSD test (p < 0.05). There was no evidence of over-dispersion given the assumption of the Poisson distribution. Genstat was used for this analysis.

### Oviposition assays

Oviposition assays were set up to assess if development in different host seeds affects subsequent female host preference (Hopkins’ host selection principle, [30]). A Petri dish (5.5 cm diam. × 1.5 cm height) was divided into quarters by a 1 cm high cross-section made of two black filter paper strips. A disc from the same type of filter paper covered the bottom of the dish to make the white eggs more visible. Three bean seeds were put in each of two opposite sectors, and three chickpea seeds were put in the other two opposite sectors. A pair of virgin *A*. *obtectus* was released into each dish, the cross division allowing free access for the beetles to the entire arena. Temperature was maintained at 20°C, and each arena was left for 5 days. There were 10 replicates for each host line. Egg numbers were summed for each seed type in each dish at the end of the experiment, and the two seed types were compared using a paired *t*-test (p < 0.05) for each female host line.

### Larval assays

To determine seed preference of 1^st^ instar larvae originating from eggs laid by 1^st^ generation females, 10 eggs per host line were placed inside a Petri dish (5.5 cm diam. × 1.5 cm height) in the middle of a space surrounded by two bean and two chickpea seeds in an alternated manner and in close proximity to each other (0.1 cm) and from the eggs (1 cm). The replication was 10 Petri dishes for the bean and chickpea lines and 7 for the chickpea/bean line. The same test was performed with eggs from females bred for over 50 generations on chickpea (10 replicate dishes, 20 eggs per replicate). The number of emerging adults was determined for each seed type, and data was analysed by paired *t*-test (p < 0.05) for each host line and number of generations.

### Electroantennography (EAG)

To test neurophysiological responses in the olfactory periphery of female *A*. *obtectus* to male odour (sex pheromone) characterising different host lines, EAG tests were conducted using the whole antennae of virgin females from the three host lines. Antennal recordings were made using Ag–AgCl glass electrodes filled with saline solution. An antenna was freshly amputated at the base from a live *A*. *obtectus* and suspended between the two electrodes. The tip of the terminal process of the antenna was removed to ensure a good contact with a high-impedance amplifier (UN-06; Ockenfels Syntech GmbH, Kirchzarten, Germany). The base of the antenna was connected to a grounded electrode. Test stimuli consisted of five virgin males from each host line in a Pasteur pipette, through which 1 mL of air was puffed, using a stimulus controller, into a humidified airstream flowing towards the antenna. Live beetles were used as stimuli [31], because EAG responses to synthetic blends are problematic to interpret owing to the different vapour pressure of constituents in the headspace inside the pipette [32]. Stimuli were presented at random order, and the absolute negative amplitude changes in response to the stimuli were recorded. A blank Pasteur pipette served as control. The experiment provided a 3 female host lines by 4 odours (three male host lines plus control) factorial treatment structure. There were 5 replicate antennae used per female host line and odour. ANOVA was used to assess the main effects and interaction between the two factors (F-tests), followed by Fisher’s LSD test (p < 0.05) to compare female responses to male odour from different host lines.

## Results

**Fig 1:**
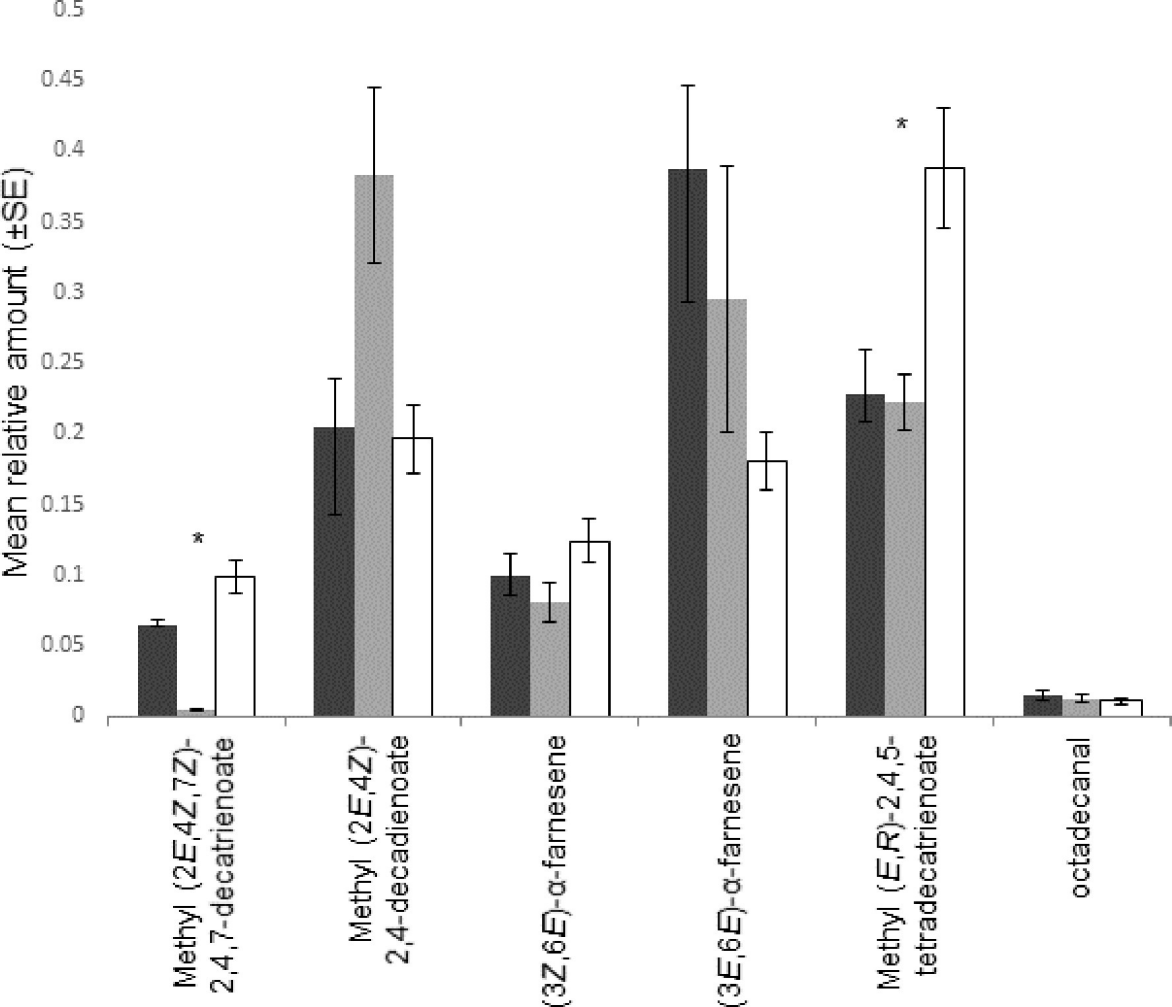
Composition of volatile extracts of male *Acanthoscelides obtectus* beetles derived from different seeds (black bars: Bean, grey bars: chickpea, empty bars: Chickpea/bean). Asterisks mark pheromone compounds the ratio of which differ significantly between host lines [n = 6 males/host line, methyl (2*E*,4*Z*,7*Z*)-2,4,7-decatrienoate p = 0.004, methyl (*E*,*R*)-2,4,5-tetradecatrienoate p = 0.006, ANOVA p = 0.05, LSD test].

GC and GC-MS analysis showed significant differences (p<0.05, F-tests) in the sex pheromone composition of *A*. *obtectus* between males originating from beans and chickpeas (Fig 1; S1 File). Intriguingly, there was an almost complete lack of methyl (2*E*,4*Z*,7*Z*)-2,4,7-decatrienoate in the headspace extract of 1^st^ generation males reared on chickpea when compared to males reared on bean. However, the emission of this compound returned after rearing 1^st^ generation chickpea beetles on bean seeds again (chickpea/bean line). The amount of methyl (*E*,*R*)-2,4,5-tetradecatrienoate doubled in males after the bean-chickpea-bean transitions.

**Fig 2:**
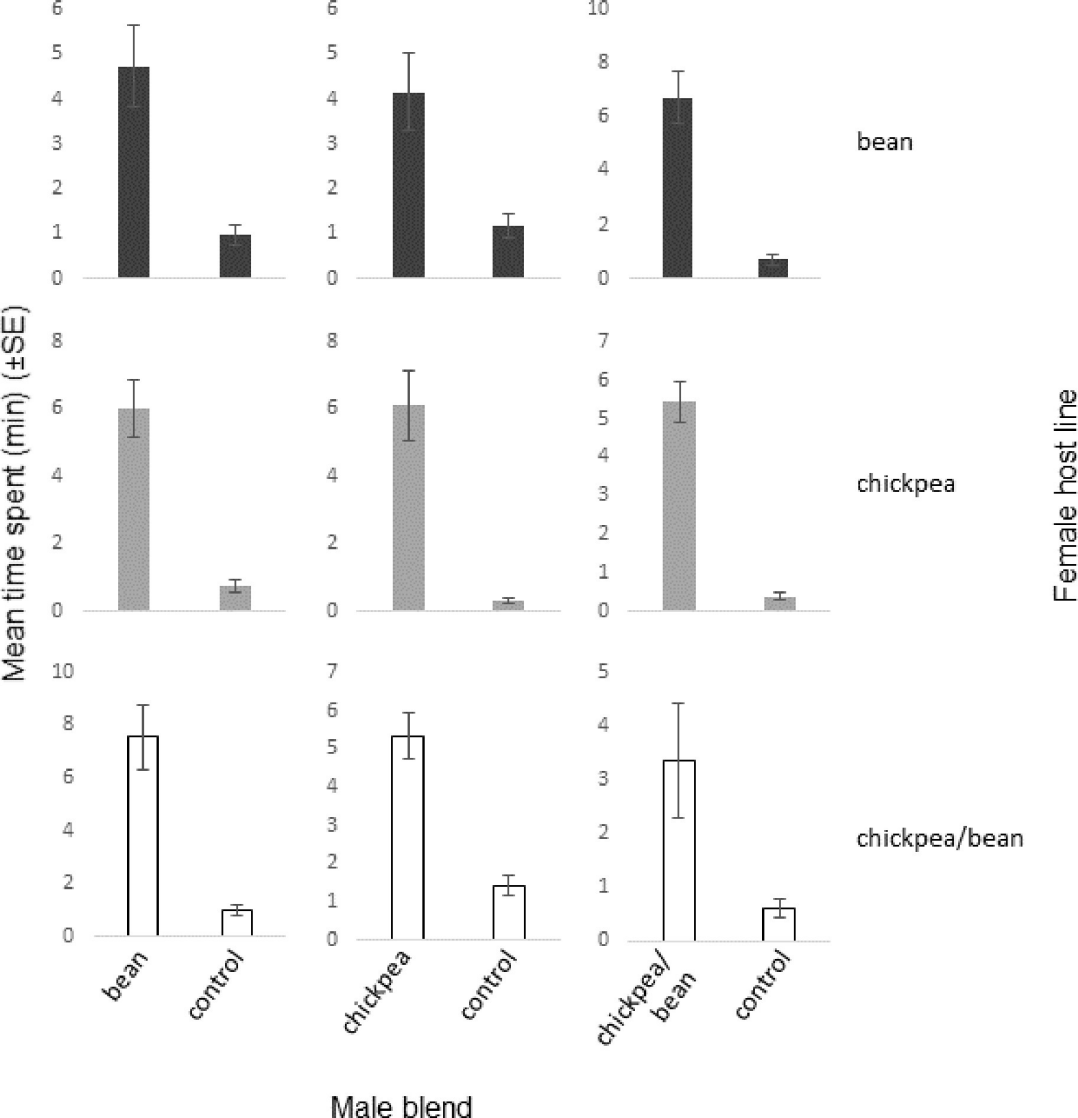
Behavioural responses of 1^st^ generation virgin *Acanthoscelides obtectus* females to synthetic male pheromone blends, representing compositions observed for the three host lines, in olfactometer bioassays. n = 10/female host line/male blend type; linear mixed model, fitted using the method of residual maximum likelihood.

**Fig 3:**
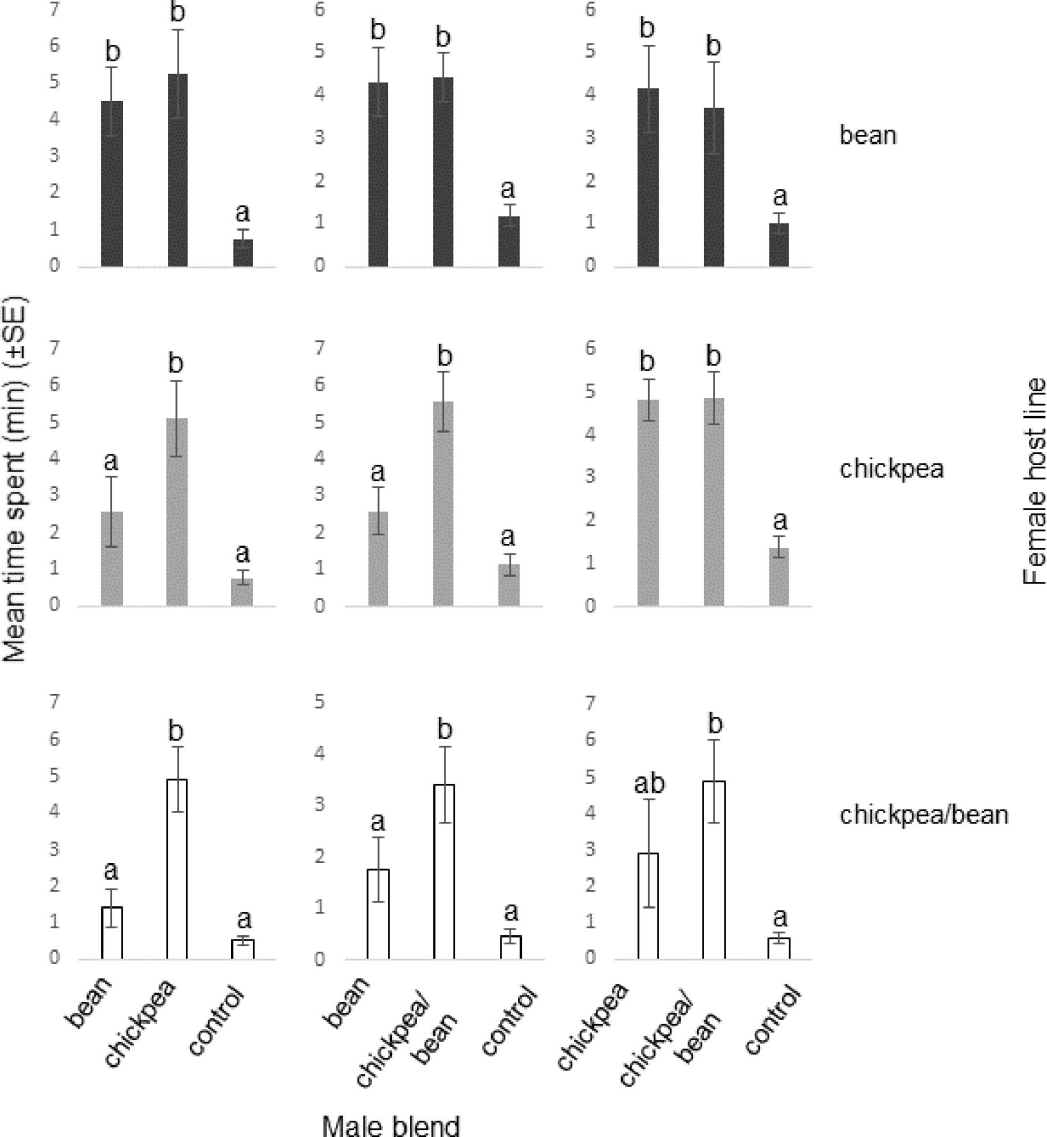
Behavioural responses of 1^st^ generation virgin *Acanthoscelides obtectus* females to synthetic male pheromone blends, representing compositions observed for the three host lines, in pairwise comparisons in olfactometer bioassays. n = 10/female host line/male blend type; linear mixed model, fitted using the method of residual maximum likelihood; columns with same letter not differing significantly (p<0.05, LSD).

Females gave strong positive behavioural responses to all types of male blend when they were presented on their own (Fig 2; Figures B-C in S2 File). When faced with pairwise choices, a host line-specific pattern emerged: females reared on beans did not discriminate between the three blend types, whereas chickpea-reared ones and those from the chickpea/bean line preferred their own type of blend to the blend of males from beans, not differentiating, however, between the chickpea and chickpea/bean types (Fig 3; Figures D-E in S2 File; the statistical analysis of the full set of single blend and pairwise blend choice assays together is given in Figures F-G in S2 File).

Still-air bioassays gave similar results to those from olfactometer assays, showing that females reared on beans did not discriminate between blends of males reared on bean and chickpea, whereas females reared on chickpea preferred the pheromone blend of chickpea-reared males compared to bean-reared males (Table 2). There was no significant difference between female numbers within each blend. There was some partial evidence of an interaction between treatment (blend) and female population (p = 0.156, Chi-squared test; Figure B in S3 File).

**Table 2:** Predicted means for the number of virgin *Acanthoscelides obtectus* females (±SE) attracted to male blends in still-air bioassays.

male blend	female host line	
	bean	chickpea
bean	1.27±0.2 (3.57) ab	0.89±0.24 (2.43) a
chickpea	1.42±0.19 (4.14) b	1.72±0.16 (5.57) b
control	0.62±0.28 (1.86) a	0.13±0.35 (1.14) a

Values are given on the natural log scale (ln) from a generalized linear model fitted to count data assuming a Poisson distribution (n = 7) (S2 File). Average (back-transformed) counts are given in brackets. Combinations in the table with the same letters are not significantly different (p<0.05, LSD), when comparing the means on the natural log scale.

**Fig 4:**
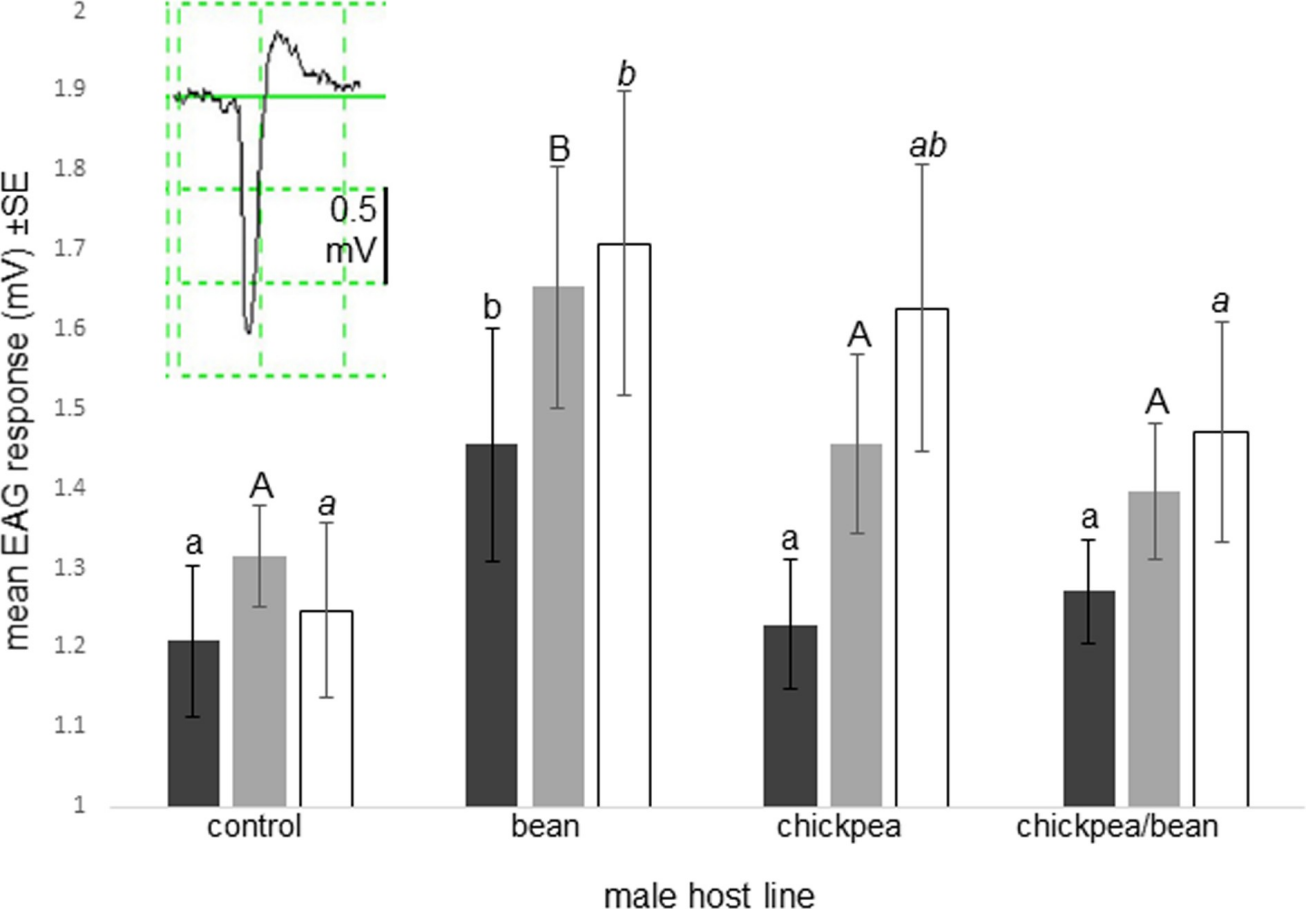
EAG responses of antennae of 1^st^ generation virgin *Acanthoscelides obtectus* females to the odour of live males from different host lines. The insert exemplifies a typical EAG response (n = 5/female host line; black bars: bean, grey bars: chickpea, empty bars: chickpea/bean; control = blank air; columns with same letter within a host line not differing significantly by ANOVA and Fisher`s LSD test, p<0.05).

Response patterns of the primary olfactory organ in female *A*. *obtectus*, the antenna, showed that independent of seed origin, females gave the strongest EAG responses to the odour of bean males, not differentiating between the other two host lines, or control (Fig 4; S4 File).

In oviposition assays, 1^st^ generation females from all host lines laid the greatest number of eggs in sectors with bean seeds as compared to sectors with chickpea seeds (Table 3). Host-searching first instar larvae, on the other hand, did not discriminate between seeds (Table 4).

**Table 3:** Results of oviposition choice tests with *Acanthoscelides obtectus*.

female host line	mean number of eggs in seed sector (±SE)		p value
	bean	chickpea	
bean	29.1±4.93	2.3±0.52	<0.001
chickpea	39.2±5.31	3.6±1.13	<0.001
chickpea/bean	24.0±2.45	5.5±1.34	<0.001

n = 10, paired *t*-tests.

**Table 4:** Seed choice by 1^st^ instar *Acanthoscelides obtectus* larvae from different host lines, measured as number of emerged adults.

host line of larvae	mean number of adults emerged (±SE)		P value
	bean	chickpea	
bean (1^st^ gen.)	0.4±0.31	1.1±0.38	0.191
chickpea (1^st^ gen.)	0.8±0.53	1.5±0.48	0.421
chickpea/bean (1^st^ gen.)*	0.29±0.29	1.14±0.51	0.248
chickpea (50^th^ gen.)**	3.1±1.05	2.1±1.05	0.572

10 eggs per replicate and n = 10 replicates per assay, paired *t*-tests.

*n = 7

**20 eggs per replicate were used in this case.

**Fig 5:**
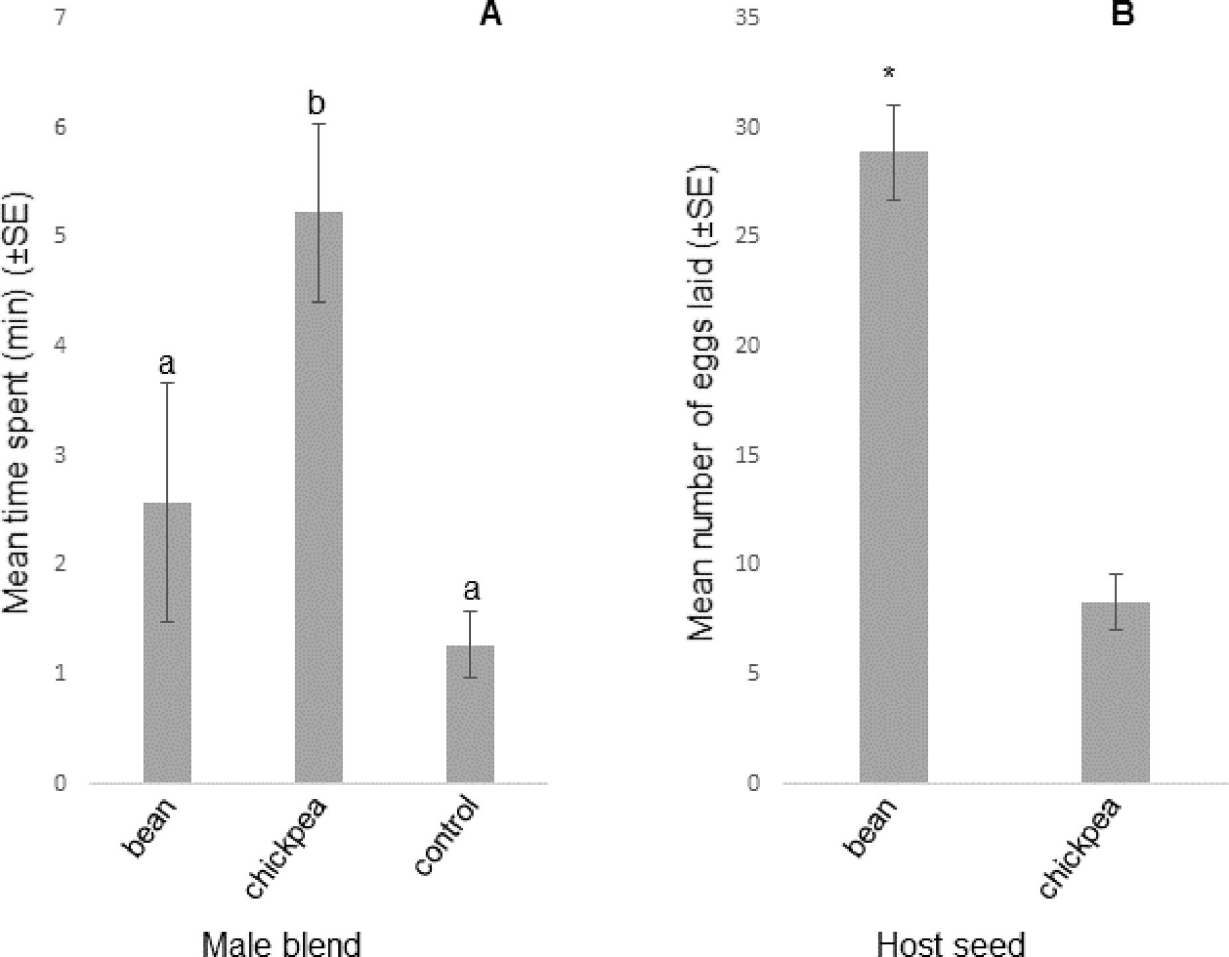
Behavioural and oviposition responses of virgin *Acanthoscelides obtectus* females reared on chickpea for 50 generations. A: Behavioural responses to synthetic male pheromone blends in olfactometer bioassays; n = 10; columns with same letter not differing significantly, linear mixed model, fitted using the method of residual maximum likelihood. B: Oviposition responses; n = 10; p<0.001, paired *t*-test; asterisk: statistical difference.

When investigating the consequences of breeding *A*. *obtectus* for 50 generations on chickpea, a strong behavioural preference for the sex pheromone blend of chickpea males over that of bean males was found in 50^th^ generation females, which was similar to that of 1^st^ generation ones (Fig 5A; Figures H-I in S2 File). However, the greatest number of eggs was laid in sectors with bean seeds (Fig 5B).

## Discussion

Artificially-induced host shifts from beans to chickpeas has been shown to cause changes in the physiology and life history traits of *A*. *obtectus* [19,20]. After the 1^st^ instar larvae have entered the seed, their development is determined by the chemical composition of the endosperm, which is reflected in the difference in life history and physiological traits between bean- and chickpea-reared *A*. *obtectus* laboratory populations. *Nasonia vitripennis* Walker wasps (Hymenoptera: Pteromalidae) benefited from an additional supply of linoleic acid in their diet, as they produced increased amounts of pheromone [33]. *N*. *vitripennis* can synthesize linoleic acid from oleic acid de novo, and males have higher pheromone titres when they develop on hosts that have been experimentally enriched in the unsaturated fatty acid precursors. We found that *A*. *obtectus* males reared on chickpea produce only trace amounts of methyl (2*E*,4*Z*,7*Z*)-2,4,7-decatrienoate, but this compound reappears in the volatile bouquet of descendant males reared on beans. The biosynthesis of methyl (2*E*,4*Z*,7*Z*)-2,4,7-decatrienoic acid, i.e., the acid part of the ester, can be rationalized either via lipoxygenase-mediated cleavage of (9*Z*,12*Z*,15*Z*)-9,12,15-octadecatrienoic acid (α-linolenic acid) or by a sequence of four β-oxidation steps and rearrangement of the same precursor [22]. As male *A*. *obtectus* do not feed as adults, the nutritional composition of the seed endosperm during larval development must determine which precursors are available in the adult stage for de novo pheromone production. As bean (*Ph*. *vulgaris*) seeds contain five times more α-linolenic acid than chickpea seeds [34], this creates a platform for new hypotheses to be formed on the biosynthetic origins of methyl (2*E*,4*Z*,7*Z*)-2,4,7-decatrienoate. Methyl (*E*,*R*)-2,4,5-tetradecatrienoate is a key component of the *A*. *obtectus* male sex pheromone [22]. Together with octadecanal, it is abundant in the cuticular wax layer of the thorax and the elytra [21], whereas the more volatile C10 methyl esters and farnesenes can only be extracted from the headspace of live males (J. Vuts, pers. observation). Methyl (*E*,*R*)-2,4,5-tetradecatrienoate is also utilised as a mate-recognition signal and as an anti-aphrodisiac, and may also be key to host line discrimination in mate-finding, as its amounts changed significantly during the chickpea-bean shift, highlighting the parsimonious use of the compound. All this points to multiple ecological functions [35] for the complex, six-component male volatile blend of *A*. *obtectus*. Considering the diversity of the genus *Acanthoscelides* (several hundred species; [36]), the pheromone may have roles for reproductive isolation encoded in its complexity.

Single-generation shift from bean to chickpea causes rapid changes in several physiological and behavioural traits in *A*. *obtectus*, including alterations in the composition of cuticular hydrocarbons, causing a certain level of sexual isolation between host lines [13,15]. As well as the altered male volatile sex pheromone composition after the bean-chickpea shift, we showed that females from the two host lines respond differently to male chemical signals. Bean-reared females did not differentiate between the bean and chickpea male pheromone blends, possibly reflecting a broad acceptance range of sex pheromone composition. Such high degree of phenotypic plasticity was also reported for other behavioural traits [19]. A novelty effect, i.e., exposure to a novel, unusual stimulus [37,38], may explain why the chickpea male blend was also found highly attractive by bean-reared females in our laboratory bioassays. However, we argue that the activity of the chickpea blend would decrease during repeated exposure because of habituation [39,40]. In contrast, females reared on chickpea showed significant preference for the male pheromone blend of their own host line, even in the first generation, indicating a plastic phenotypic process in action in response to the new environment. Their response profiles may be attributed to a high excitatory state of the central nervous system (CNS) [41], causing increased reactivity to their own host line blend (sensitisation). Chickpea-reared *A*. *obtectus* was described as behaviourally less plastic [19], which, in the light of data presented here, may also include their sexual behaviour. According to West-Eberhard ([42], p. 253), “…the probability of producing a favourable variant (in a new or challenging situation at a particular time) is greater for behaviour (and other relatively plastic traits) than for morphology.” Also, “The key to the evolutionary role of behaviour, however, is not lability alone, but a combination of lability and the consistency with which given behaviors occur in given conditions: a genetic (evolutionary) response to selection depends on a repeated association, under selection, of a particular phenotype… and a particular fitness-affecting condition…” It is argued that adaptive behavioural plasticity evolves more easily than adaptive morphological plasticity, due to the greater abundance of potential cues for regulating the expression of an immediate (behavioural) adaptive response [42]. Thus, (olfactory) sensitisation via continuous reinforcement is suggested to be maintained in *A*. *obtectus* chickpea females breeding on chickpea by regular exposure to the male pheromone (environmental conditioning). This new phenotype can then become fixed in the experimental environment (chickpea host) by epigenetic inheritance [43]. However, the phenotype sensitised to the chickpea male pheromone can only be maintained in F1 chickpea/bean females if it has an adaptive value in the new environment. To show that the narrowly tuned response profile of chickpea beetles can be inherited by the F1 generation developing on a new host (bean), cross-testing studies will be necessary.

The oviposition assays in this paper indicate no change in host plant preference in any of the first-generation host lines, or in beetles that are continuously reared on chickpea. This contradicts previous work, which found that chickpea-reared females laid more eggs on chickpea than on bean [44]. Experience can narrow down the inherited (potential) host range of certain polyphagous larvae, i.e., feeding on one host species can reduce the acceptability of other hosts in later instars (induced preference; [45]). However, only a few examples of Hopkins’ host selection principle exist [46–48]), and the general agreement is that larval experience does not influence oviposition behaviour ([30,49] and references therein). Also, again contrary to previous work [44], there seems to be no correlation between female oviposition preference and larval performance in *A*. *obtectus*. Females of all host lines preferred to lay eggs on bean seeds, whereas larvae entered both beans and chickpeas with equal frequency, measured as adult emergence (performance). No obvious correlation was found between maternal host preference and F1 performance either, concluding that the female’s oviposition decisions are suboptimal [50]. She most probably assesses the physico-chemical characteristics of the seed testa before egg-laying, which, however, does not convey information about the endosperm, the primary factor influencing larval survival. First-instar larvae entering the seeds chew through the seed coat and then spit it out, a behaviour performed possibly to avoid toxic lignin, polyphenolic or condensed tannin compounds present in the testa [51]. Being mobile, they can actively choose between host plants [52] and thus may increase their chance of survival if given the choice between seeds. Apart from the genetically determined host range, there is no information exchange between the female and the L1 larvae about the suitability of substrates, resulting in the lack of positive correlation between female oviposition preference and larval performance. Thus, the preference-performance hypothesis [53], i.e., female insects preferentially oviposit on plants that maximise the survival and performance of their larvae, has no support in *A*. *obtectus*.

It was suggested [4,54] that behavioural changes can be induced in phytophagous insects by mutations that alter chemoreceptor sites, facilitating diversification on to new host plants. Recent studies highlight the key role of both the central and peripheral nervous system in semiochemical-based herbivore host specialisation [55,56] and the importance of mutations affecting metabolic pathways and the olfactory system in shaping host specialisation processes [57]. Host shift in phytophagous insects is suggested to be characterised by the loss of recognising the original host plant (‘switch-over’), and is not an extension of host range [58]. It is argued that a loss of sensitivity to deterrents of the potential new host is a prerequisite for host switch [59]. However, others suggest the terminology of ‘host range expansion’ for cases when a population colonizes a new host plant but continues to use previous host plants [60]. It is not possible to determine which process may be true for *A*. *obtectus*, simply because the phenomenon has only been studied under experimental situations, which was pointed out before [61]. It was shown that laboratory-reared *A*. *obtectus* larvae can develop in suboptimal host seeds, such as chickpea [62–64]. However, this is reported also in natural populations in chickpea-producing areas [65–67]. Where beans and chickpeas are cultivated and stored in close proximity, for example in India, the Middle East or East Africa, host shift to chickpea may happen under natural circumstances. Thus, studies presented in this paper must be extended to such sympatric wild populations.

Since antennae of both first-generation *A*. *obtectus* females and those reared on chickpea continuously gave very similar electrophysiological (EAG) responses to the odour of males across the host lines included in this study, we conclude that plastic processes in the CNS, but not in the peripheral nervous system, determine female behaviour towards male pheromones. The modulatory effect of the environment, e.g., starvation, on EAG responses has been shown in a number of insect species [68], but such peripheral sensory modifications do not appear to be involved in *A*. *obtectus* chemical communication under host shifts. Although significant alterations were observed in chemical signalling immediately after shift from one host to another (our hypothesis 1), which were maintained over generations in chickpea lines (hypothesis 2), there is no support for hypothesis 3 (altered chemical communication facilitates host shift that may eventually lead to reproductive isolation) from our studies. Both the behavioural and electrophysiological data suggest that the more common bean line would assimilate rarer populations of the chickpea line in sympatry, because bean-reared females do not discriminate between host line pheromone blends. Also, because the level of male mate discrimination reduces after shift from bean to chickpea [13], narrow mate preference of chickpea females would be counteracted by non-discriminatory chickpea males. In addition, the effect of assortative mating in bean-reared males [13] would be similarly weakened by non-discriminatory bean females.

Assuming that the hypothesis by Quental et al. [11] holds true, i.e., newly acquired compounds sequestered from alternative hosts influence female mate preferences and drives sexual isolation and host shift, bean-reared females would be expected to select for their own host line pheromone; bean and chickpea lines should embody plastic changes occurring in the CNS into morphological changes in the olfactory periphery at the neuronal level (although morphological changes are rarer to acquire than behavioural; [42]); oviposition substrate choices should change accordingly. According to the hierarchy-threshold model [69], each plant species the insect encounters has an intrinsic acceptability rank, and if stimuli received from the plant exceed a certain threshold, the plant will be accepted. The model predicts “the maintenance of significant genetic variance for acceptability of hosts which are not used in nature, since this genetic variance is not subject to selection” ([69], p.61). Thus, oviposition on an acceptable non-host in *A*. *obtectus* may be a result of changes in the hierarchy threshold for that plant (i.e., chickpea), a phenomenon also indicated for *Vigna radiata* (L.) R. Wilczek/*Vigna unguiculata* L. and *Callosobruchus maculatus* Fabr., another legume-bruchid system [70]. Host shift in field *A*. *obtectus* populations would require a fine balance among several factors, including the female’s ability to recognise the pericarp and accept the fruit of the acceptable non-host. As larvae can develop in chickpea but larval experience does not influence oviposition choice, the process breaks at every egg-laying event. How pheromones may facilitate host shift remains to be answered, for which studies with individuals from sympatric wild populations will be necessary, as outlined above. For *A*. *obtectus* and other bruchids, such experiments may also provide key information for the design of novel, semiochemical-based management strategies [71].

## Supporting information

S1 Fig Age-specific release of *Acanthoscelides obtectus* sex pheromone components by virgin males from the bean line. Raw data included.
(DOCX)

S1 File Raw data for pheromone release from males from different host lines. (DOCX)

S2 File Olfactometer data and analysis. (DOCX)

S3 File Still-air bioassay data and analysis. (DOCX)

S4 File EAG data for females from different host lines. (DOCX)
