# Supplementary material for: Host shift induces changes in mate choice of the seed predator Acanthoscelides obtectus via altered chemical signalling
Source: PLoS One. 2018 Nov 14;13(11):e0206144. doi: 10.1371/journal.pone.0206144 (PMC6235263; doi:10.1371/journal.pone.0206144)
Supplement: S1 Fig — Raw data included. (DOCX) [file pone.0206144.s001.docx]

**Host shift induces changes in mate choice of the seed predator *Acanthoscelides obtectus* via altered chemical signalling**

József Vuts, Christine M. Woodcock, Lisa König, Stephen J. Powers, John A. Pickett, Árpád Szentesi, Michael A. Birkett


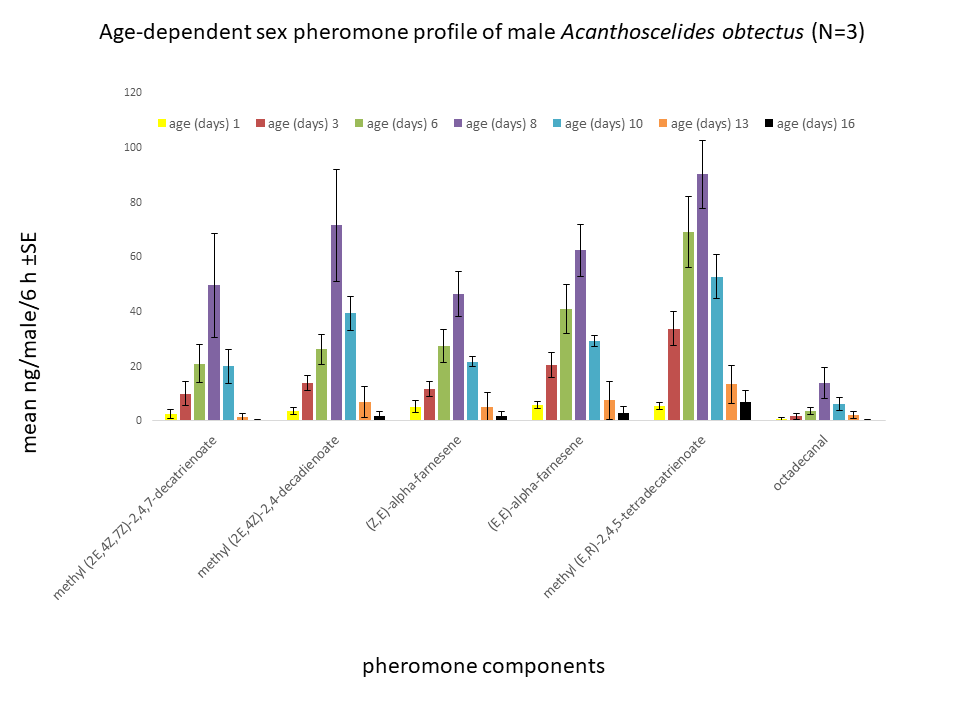


**S1 Fig.**

Raw data (values=ng compound/male/6 h):

| replicate | Compounds | age (days) | | | | | | |
| --- | --- | --- | --- | --- | --- | --- | --- | --- |
|  |  | 1 | 3 | 6 | 8 | 10 | 13 | 16 |
| 1 | methyl (2*E*,4*Z*,7*Z*)-2,4,7-decatrienoate | 1.40 | 5.40 | 10.63 | 11.59 | 7.46 | 3.98 | 0.69 |
| 1 | methyl (2*E*,4*Z*)-2,4-decadienoate | 4.74 | 16.57 | 28.24 | 30.90 | 29.24 | 18.41 | 4.95 |
| 1 | (*Z,E*)-alpha-farnesene | 3.64 | 15.80 | 27.94 | 30.20 | 20.47 | 15.27 | 5.36 |
| 1 | (*E,E*)-alpha-farnesene | 6.00 | 24.13 | 43.33 | 44.51 | 29.78 | 21.64 | 8.03 |
| 1 | methyl (*E*,*R*)-2,4,5-tetradecatrienoate | 3.84 | 23.11 | 54.07 | 65.69 | 36.99 | 27.31 | 15.58 |
| 1 | octadecanal | 0.11 | 0.51 | 2.27 | 3.11 | 1.30 | 1.62 | 0.80 |
| 2 | methyl (2*E*,4*Z*,7*Z*)-2,4,7-decatrienoate | 5.65 | 18.66 | 34.09 | 65.57 | 28.14 | 0.26 | 0.11 |
| 2 | methyl (2*E*,4*Z*)-2,4-decadienoate | 4.99 | 16.96 | 34.68 | 88.12 | 51.06 | 1.51 | 0.74 |
| 2 | (*Z,E*)-alpha-farnesene | 9.43 | 12.37 | 37.40 | 57.29 | 25.16 | 0.22 | 0.02 |
| 2 | (*E,E*)-alpha-farnesene | 8.26 | 26.09 | 54.85 | 77.37 | 32.44 | 0.46 | 0.29 |
| 2 | methyl (*E*,*R*)-2,4,5-tetradecatrienoate | 7.98 | 44.41 | 94.76 | 97.65 | 58.55 | 7.29 | 3.45 |
| 2 | octadecanal | 2.11 | 3.82 | 6.26 | 22.40 | 8.44 | 0.50 | 0.34 |
| 3 | methyl (2*E*,4*Z*,7*Z*)-2,4,7-decatrienoate | 0.44 | 5.87 | 18.01 | 71.65 | 24.25 | 0.29 | 0.06 |
| 3 | methyl (2*E*,4*Z*)-2,4-decadienoate | 1.36 | 8.13 | 15.89 | 95.48 | 37.59 | 1.29 | 0.27 |
| 3 | (*Z,E*)-alpha-farnesene | 2.55 | 6.77 | 16.57 | 51.54 | 19.61 | 0.20 | 0.04 |
| 3 | (*E,E*)-alpha-farnesene | 3.72 | 11.13 | 24.43 | 65.28 | 25.70 | 0.38 | 0.17 |
| 3 | methyl (*E*,*R*)-2,4,5-tetradecatrienoate | 4.94 | 33.64 | 58.60 | 106.99 | 62.84 | 5.44 | 1.33 |
| 3 | octadecanal | 0.19 | 0.87 | 2.29 | 15.65 | 9.04 | 4.61 | 0.22 |
