# Supplementary material for: Host shift induces changes in mate choice of the seed predator Acanthoscelides obtectus via altered chemical signalling
Source: PLoS One. 2018 Nov 14;13(11):e0206144. doi: 10.1371/journal.pone.0206144 (PMC6235263; doi:10.1371/journal.pone.0206144)
Supplement: S1 File — (DOCX) [file pone.0206144.s002.docx]

**Host shift induces changes in mate choice of the seed predator *Acanthoscelides obtectus* via altered chemical signalling**

József Vuts, Christine M. Woodcock, Lisa König, Stephen J. Powers, John A. Pickett, Árpád Szentesi, Michael A. Birkett

Raw data: GC area % of the six male pheromone components

| male host line | methyl (2*E*,4*Z*,7*Z*)-2,4,7-decatrienoate | methyl (2*E*,4*Z*)-2,4-decadienoate | (3*Z*,6*E*)-α-farnesene | (3*E*,6*E*)-α-farnesene | methyl (*E,R*)-2,4,5-tetradecatrienoate | octadecanal |
| --- | --- | --- | --- | --- | --- | --- |
| chickpea | 0.004651 | 0.413419 | 0.089443 | 0.218544 | 0.256076 | 0.017867 |
| chickpea | 0.006884 | 0.428866 | 0.091015 | 0.240361 | 0.214484 | 0.01839 |
| chickpea | 0.005266 | 0.413466 | 0.078808 | 0.259724 | 0.233391 | 0.009345 |
| chickpea | 0 | 0.081422 | 0.017867 | 0.754807 | 0.145905 | 0 |
| chickpea | 0.008714 | 0.504425 | 0.115185 | 0.164686 | 0.197843 | 0.009147 |
| chickpea | 0.004419 | 0.454392 | 0.095214 | 0.136382 | 0.287736 | 0.021858 |
| bean | 0.055571 | 0.358943 | 0.093807 | 0.224852 | 0.258692 | 0.008136 |
| bean | 0.074576 | 0.201472 | 0.165905 | 0.371278 | 0.168331 | 0.018438 |
| bean | 0.074335 | 0.157702 | 0.081469 | 0.406366 | 0.268569 | 0.011559 |
| bean | 0.072644 | 0.20512 | 0.118001 | 0.310309 | 0.270593 | 0.023334 |
| bean | 0.05065 | 0.195737 | 0.074683 | 0.34924 | 0.301297 | 0.028393 |
| bean | 0.06004 | 0.108346 | 0.065759 | 0.659188 | 0.106667 | 0 |
| chickpea/bean | 0.100774 | 0.151238 | 0.139517 | 0.208986 | 0.383628 | 0.015857 |
| chickpea/bean | 0.127412 | 0.156279 | 0.069815 | 0.110311 | 0.532651 | 0.003532 |
| chickpea/bean | 0.079063 | 0.240837 | 0.114535 | 0.161988 | 0.397111 | 0.006467 |
| chickpea/bean | 0.06503 | 0.265957 | 0.157709 | 0.224223 | 0.272604 | 0.014477 |
| chickpea/bean | 0 | 0 | 0 | 0 | 1 | 0 |
| chickpea/bean | 0.12126 | 0.166717 | 0.14054 | 0.200089 | 0.356702 | 0.014692 |
