# Supplementary material for: Host shift induces changes in mate choice of the seed predator Acanthoscelides obtectus via altered chemical signalling
Source: PLoS One. 2018 Nov 14;13(11):e0206144. doi: 10.1371/journal.pone.0206144 (PMC6235263; doi:10.1371/journal.pone.0206144)
Supplement: S2 File — (DOCX) [file pone.0206144.s003.docx]

**Host shift induces changes in mate choice of the seed predator *Acanthoscelides obtectus* via altered chemical signalling**

József Vuts, Christine M. Woodcock, Lisa König, Stephen J. Powers, John A. Pickett, Árpád Szentesi, Michael A. Birkett

**Olfactometer tests**


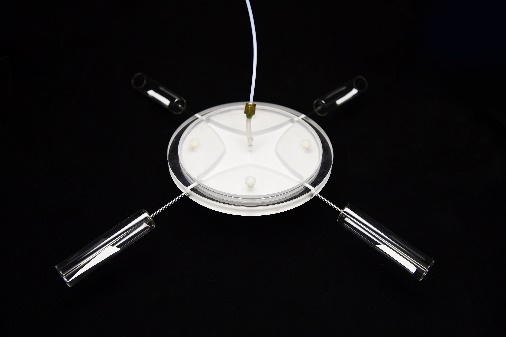


Fig. A

**Section 1. Single blend assays**

Raw data (values=time in minute)

| **Bean females to bean blend** | | |  |
| --- | --- | --- | --- |
| replicate | Area | Treatment | time spent |
| 1 | 1 | Tr1 | 0.93 |
| 1 | 2 | Control | 0 |
| 1 | 3 | Control | 0 |
| 1 | 4 | Control | 1.52 |
| 2 | 1 | Tr1 | 8.2 |
| 2 | 2 | Control | 0 |
| 2 | 3 | Control | 0 |
| 2 | 4 | Control | 0.68 |
| 3 | 1 | Tr1 | 3.13 |
| 3 | 2 | Control | 0 |
| 3 | 3 | Control | 0 |
| 3 | 4 | Control | 0.12 |
| 4 | 1 | Tr1 | 4.12 |
| 4 | 2 | Control | 3.05 |
| 4 | 3 | Control | 1.28 |
| 4 | 4 | Control | 0.9 |
| 5 | 1 | Tr1 | 5.97 |
| 5 | 2 | Control | 1.08 |
| 5 | 3 | Control | 0.62 |
| 5 | 4 | Control | 0 |
| 6 | 1 | Tr1 | 2.62 |
| 6 | 2 | Control | 0 |
| 6 | 3 | Control | 2.88 |
| 6 | 4 | Control | 5.07 |
| 7 | 1 | Tr1 | 4.17 |
| 7 | 2 | Control | 3 |
| 7 | 3 | Control | 0 |
| 7 | 4 | Control | 0 |
| 8 | 1 | Tr1 | 1.45 |
| 8 | 2 | Control | 0.77 |
| 8 | 3 | Control | 1.27 |
| 8 | 4 | Control | 2.9 |
| 9 | 1 | Tr1 | 8.27 |
| 9 | 2 | Control | 0.72 |
| 9 | 3 | Control | 0.4 |
| 9 | 4 | Control | 2.44 |
| 10 | 1 | Tr1 | 8.48 |
| 10 | 2 | Control | 0.02 |
| 10 | 3 | Control | 0.77 |
| 10 | 4 | Control | 0 |

| **Bean females to chickpea blend** | | | |
| --- | --- | --- | --- |
| replicate | Area | Treatment | time spent |
| 1 | 1 | Tr1 | 3.9 |
| 1 | 2 | Control | 0 |
| 1 | 3 | Control | 0.1 |
| 1 | 4 | Control | 1.57 |
| 2 | 1 | Tr1 | 0 |
| 2 | 2 | Control | 6.92 |
| 2 | 3 | Control | 0 |
| 2 | 4 | Control | 0 |
| 3 | 1 | Tr1 | 5.3 |
| 3 | 2 | Control | 1.33 |
| 3 | 3 | Control | 0.33 |
| 3 | 4 | Control | 1.48 |
| 4 | 1 | Tr1 | 5.13 |
| 4 | 2 | Control | 0 |
| 4 | 3 | Control | 0 |
| 4 | 4 | Control | 0.15 |
| 5 | 1 | Tr1 | 7.02 |
| 5 | 2 | Control | 0 |
| 5 | 3 | Control | 0 |
| 5 | 4 | Control | 0.03 |
| 6 | 1 | Tr1 | 8.55 |
| 6 | 2 | Control | 0 |
| 6 | 3 | Control | 0 |
| 6 | 4 | Control | 2.28 |
| 7 | 1 | Tr1 | 0 |
| 7 | 2 | Control | 4.83 |
| 7 | 3 | Control | 0.75 |
| 7 | 4 | Control | 1 |
| 8 | 1 | Tr1 | 4.88 |
| 8 | 2 | Control | 1.63 |
| 8 | 3 | Control | 1.02 |
| 8 | 4 | Control | 1.6 |
| 9 | 1 | Tr1 | 3.95 |
| 9 | 2 | Control | 2.03 |
| 9 | 3 | Control | 1.73 |
| 9 | 4 | Control | 1.58 |
| 10 | 1 | Tr1 | 2.8 |
| 10 | 2 | Control | 1.47 |
| 10 | 3 | Control | 1.25 |
| 10 | 4 | Control | 2.45 |

| **Bean females to chickpea-bean blend** | | | |
| --- | --- | --- | --- |
| replicate | Area | Treatment | time spent |
| 1 | 1 | Tr1 | 8.37 |
| 1 | 2 | Control | 1.27 |
| 1 | 3 | Control | 0.9 |
| 1 | 4 | Control | 0 |
| 2 | 1 | Tr1 | 7.17 |
| 2 | 2 | Control | 0 |
| 2 | 3 | Control | 0 |
| 2 | 4 | Control | 0 |
| 3 | 1 | Tr1 | 3.08 |
| 3 | 2 | Control | 0 |
| 3 | 3 | Control | 0.9 |
| 3 | 4 | Control | 0 |
| 4 | 1 | Tr1 | 2.1 |
| 4 | 2 | Control | 5.08 |
| 4 | 3 | Control | 1.17 |
| 4 | 4 | Control | 1.33 |
| 5 | 1 | Tr1 | 7.92 |
| 5 | 2 | Control | 0.53 |
| 5 | 3 | Control | 0 |
| 5 | 4 | Control | 0 |
| 6 | 1 | Tr1 | 6.92 |
| 6 | 2 | Control | 0.35 |
| 6 | 3 | Control | 0 |
| 6 | 4 | Control | 0 |
| 7 | 1 | Tr1 | 6.18 |
| 7 | 2 | Control | 2.82 |
| 7 | 3 | Control | 0 |
| 7 | 4 | Control | 0 |
| 8 | 1 | Tr1 | 13.17 |
| 8 | 2 | Control | 0.85 |
| 8 | 3 | Control | 0.17 |
| 8 | 4 | Control | 0.05 |
| 9 | 1 | Tr1 | 5.7 |
| 9 | 2 | Control | 1.7 |
| 9 | 3 | Control | 0.5 |
| 9 | 4 | Control | 0.93 |
| 10 | 1 | Tr1 | 6.58 |
| 10 | 2 | Control | 0.75 |
| 10 | 3 | Control | 1.17 |
| 10 | 4 | Control | 0.58 |

| **Chickpea females to bean blend** | | | |
| --- | --- | --- | --- |
| replicate | Area | Treatment | time spent |
| 1 | 1 | Tr1 | 10.13 |
| 1 | 2 | Control | 0 |
| 1 | 3 | Control | 0 |
| 1 | 4 | Control | 0 |
| 2 | 1 | Tr1 | 6.97 |
| 2 | 2 | Control | 0 |
| 2 | 3 | Control | 1.03 |
| 2 | 4 | Control | 1.62 |
| 3 | 1 | Tr1 | 9.82 |
| 3 | 2 | Control | 0 |
| 3 | 3 | Control | 0.15 |
| 3 | 4 | Control | 0.82 |
| 4 | 1 | Tr1 | 7.6 |
| 4 | 2 | Control | 0.17 |
| 4 | 3 | Control | 0 |
| 4 | 4 | Control | 0.8 |
| 5 | 1 | Tr1 | 2.7 |
| 5 | 2 | Control | 3.92 |
| 5 | 3 | Control | 1.22 |
| 5 | 4 | Control | 1.58 |
| 6 | 1 | Tr1 | 3.65 |
| 6 | 2 | Control | 0.75 |
| 6 | 3 | Control | 0 |
| 6 | 4 | Control | 2.88 |
| 7 | 1 | Tr1 | 3.03 |
| 7 | 2 | Control | 0 |
| 7 | 3 | Control | 0 |
| 7 | 4 | Control | 0.85 |
| 8 | 1 | Tr1 | 6.03 |
| 8 | 2 | Control | 2.5 |
| 8 | 3 | Control | 0.93 |
| 8 | 4 | Control | 0.37 |
| 9 | 1 | Tr1 | 4.23 |
| 9 | 2 | Control | 0 |
| 9 | 3 | Control | 0 |
| 9 | 4 | Control | 0 |
| 10 | 1 | Tr1 | 6.08 |
| 10 | 2 | Control | 2.22 |
| 10 | 3 | Control | 0 |
| 10 | 4 | Control | 0 |

| **Chickpea females to chickpea blend** | | | |
| --- | --- | --- | --- |
| replicate | Area | Treatment | time spent |
| 1 | 1 | Tr1 | 1.55 |
| 1 | 2 | Control | 0 |
| 1 | 3 | Control | 0.12 |
| 1 | 4 | Control | 0 |
| 2 | 1 | Tr1 | 6.43 |
| 2 | 2 | Control | 0.37 |
| 2 | 3 | Control | 0.08 |
| 2 | 4 | Control | 0 |
| 3 | 1 | Tr1 | 10.95 |
| 3 | 2 | Control | 0.12 |
| 3 | 3 | Control | 0 |
| 3 | 4 | Control | 0 |
| 4 | 1 | Tr1 | 7.43 |
| 4 | 2 | Control | 0 |
| 4 | 3 | Control | 0 |
| 4 | 4 | Control | 0.1 |
| 5 | 1 | Tr1 | 7.53 |
| 5 | 2 | Control | 1.67 |
| 5 | 3 | Control | 0 |
| 5 | 4 | Control | 0.02 |
| 6 | 1 | Tr1 | 8.58 |
| 6 | 2 | Control | 0 |
| 6 | 3 | Control | 0.77 |
| 6 | 4 | Control | 0.68 |
| 7 | 1 | Tr1 | 9.38 |
| 7 | 2 | Control | 0 |
| 7 | 3 | Control | 0.45 |
| 7 | 4 | Control | 0.83 |
| 8 | 1 | Tr1 | 3.03 |
| 8 | 2 | Control | 0.15 |
| 8 | 3 | Control | 0 |
| 8 | 4 | Control | 0 |
| 9 | 1 | Tr1 | 4.68 |
| 9 | 2 | Control | 0 |
| 9 | 3 | Control | 0.13 |
| 9 | 4 | Control | 0.03 |
| 10 | 1 | Tr1 | 1.62 |
| 10 | 2 | Control | 1.58 |
| 10 | 3 | Control | 1.18 |
| 10 | 4 | Control | 1.05 |

| **Chickpea females to chickpea-bean blend** | | | |
| --- | --- | --- | --- |
| replicate | Area | Treatment | time spent |
| 1 | 1 | Tr1 | 4.47 |
| 1 | 2 | Control | 0.52 |
| 1 | 3 | Control | 0 |
| 1 | 4 | Control | 0 |
| 2 | 1 | Tr1 | 4.87 |
| 2 | 2 | Control | 0.25 |
| 2 | 3 | Control | 0.13 |
| 2 | 4 | Control | 1.4 |
| 3 | 1 | Tr1 | 4.55 |
| 3 | 2 | Control | 0.85 |
| 3 | 3 | Control | 0 |
| 3 | 4 | Control | 0.25 |
| 4 | 1 | Tr1 | 6.38 |
| 4 | 2 | Control | 0.52 |
| 4 | 3 | Control | 0 |
| 4 | 4 | Control | 0 |
| 5 | 1 | Tr1 | 6.28 |
| 5 | 2 | Control | 1.92 |
| 5 | 3 | Control | 0 |
| 5 | 4 | Control | 0 |
| 6 | 1 | Tr1 | 5.63 |
| 6 | 2 | Control | 0.62 |
| 6 | 3 | Control | 0 |
| 6 | 4 | Control | 0 |
| 7 | 1 | Tr1 | 9.58 |
| 7 | 2 | Control | 0 |
| 7 | 3 | Control | 0 |
| 7 | 4 | Control | 0 |
| 8 | 1 | Tr1 | 3.98 |
| 8 | 2 | Control | 0.43 |
| 8 | 3 | Control | 0.55 |
| 8 | 4 | Control | 0.82 |
| 9 | 1 | Tr1 | 4.18 |
| 9 | 2 | Control | 0 |
| 9 | 3 | Control | 0 |
| 9 | 4 | Control | 1.4 |
| 10 | 1 | Tr1 | 4.53 |
| 10 | 2 | Control | 0.98 |
| 10 | 3 | Control | 0.53 |
| 10 | 4 | Control | 0 |

| **Chickpea-bean females to bean blend** | | | |
| --- | --- | --- | --- |
| replicate | Area | Treatment | time spent |
| 1 | 1 | Tr1 | 10.5 |
| 1 | 2 | Control | 0.6 |
| 1 | 3 | Control | 0 |
| 1 | 4 | Control | 1.3 |
| 2 | 1 | Tr1 | 10.83 |
| 2 | 2 | Control | 1.12 |
| 2 | 3 | Control | 0 |
| 2 | 4 | Control | 0 |
| 3 | 1 | Tr1 | 6.62 |
| 3 | 2 | Control | 2.83 |
| 3 | 3 | Control | 0.65 |
| 3 | 4 | Control | 1.22 |
| 4 | 1 | Tr1 | 12.25 |
| 4 | 2 | Control | 0.7 |
| 4 | 3 | Control | 0.1 |
| 4 | 4 | Control | 0 |
| 5 | 1 | Tr1 | 5.53 |
| 5 | 2 | Control | 0.7 |
| 5 | 3 | Control | 3.73 |
| 5 | 4 | Control | 0.75 |
| 6 | 1 | Tr1 | 4.98 |
| 6 | 2 | Control | 1.85 |
| 6 | 3 | Control | 1.97 |
| 6 | 4 | Control | 2.98 |
| 7 | 1 | Tr1 | 12.25 |
| 7 | 2 | Control | 0.08 |
| 7 | 3 | Control | 0 |
| 7 | 4 | Control | 0.08 |
| 8 | 1 | Tr1 | 3.57 |
| 8 | 2 | Control | 2.43 |
| 8 | 3 | Control | 1.8 |
| 8 | 4 | Control | 2.93 |
| 9 | 1 | Tr1 | 8.25 |
| 9 | 2 | Control | 1.42 |
| 9 | 3 | Control | 0 |
| 9 | 4 | Control | 0 |
| 10 | 1 | Tr1 | 0.72 |
| 10 | 2 | Control | 0.73 |
| 10 | 3 | Control | 0 |
| 10 | 4 | Control | 0.22 |

| **Chickpea-bean females to chickpea blend** | | | |
| --- | --- | --- | --- |
| replicate | Area | Treatment | time spent |
| 1 | 1 | Tr1 | 5.88 |
| 1 | 2 | Control | 1.57 |
| 1 | 3 | Control | 0 |
| 1 | 4 | Control | 0 |
| 2 | 1 | Tr1 | 3.25 |
| 2 | 2 | Control | 1.85 |
| 2 | 3 | Control | 0.37 |
| 2 | 4 | Control | 0 |
| 3 | 1 | Tr1 | 7.57 |
| 3 | 2 | Control | 1.43 |
| 3 | 3 | Control | 0.03 |
| 3 | 4 | Control | 0.03 |
| 4 | 1 | Tr1 | 3.73 |
| 4 | 2 | Control | 0 |
| 4 | 3 | Control | 1.23 |
| 4 | 4 | Control | 2.63 |
| 5 | 1 | Tr1 | 6.3 |
| 5 | 2 | Control | 3.93 |
| 5 | 3 | Control | 2.13 |
| 5 | 4 | Control | 1.27 |
| 6 | 1 | Tr1 | 8.73 |
| 6 | 2 | Control | 3.13 |
| 6 | 3 | Control | 1 |
| 6 | 4 | Control | 0.3 |
| 7 | 1 | Tr1 | 4.2 |
| 7 | 2 | Control | 2.53 |
| 7 | 3 | Control | 1.47 |
| 7 | 4 | Control | 3.9 |
| 8 | 1 | Tr1 | 6.03 |
| 8 | 2 | Control | 0 |
| 8 | 3 | Control | 0 |
| 8 | 4 | Control | 4.45 |
| 9 | 1 | Tr1 | 4.68 |
| 9 | 2 | Control | 0 |
| 9 | 3 | Control | 0 |
| 9 | 4 | Control | 0.15 |
| 10 | 1 | Tr1 | 3.05 |
| 10 | 2 | Control | 4 |
| 10 | 3 | Control | 3.63 |
| 10 | 4 | Control | 1.83 |

| **Chickpea-bean females to chickpea-bean blend** | | | |
| --- | --- | --- | --- |
| replicate | Area | Treatment | time spent |
| 1 | 1 | Tr1 | 10.45 |
| 1 | 2 | Control | 0 |
| 1 | 3 | Control | 0.05 |
| 1 | 4 | Control | 0.75 |
| 2 | 1 | Tr1 | 1.22 |
| 2 | 2 | Control | 1.98 |
| 2 | 3 | Control | 3.68 |
| 2 | 4 | Control | 0.29 |
| 3 | 1 | Tr1 | 2.82 |
| 3 | 2 | Control | 2.22 |
| 3 | 3 | Control | 1.5 |
| 3 | 4 | Control | 0 |
| 4 | 1 | Tr1 | 0 |
| 4 | 2 | Control | 0.32 |
| 4 | 3 | Control | 1.27 |
| 4 | 4 | Control | 0 |
| 5 | 1 | Tr1 | 0.48 |
| 5 | 2 | Control | 0.47 |
| 5 | 3 | Control | 0 |
| 5 | 4 | Control | 0 |
| 6 | 1 | Tr1 | 2.68 |
| 6 | 2 | Control | 1.1 |
| 6 | 3 | Control | 0.7 |
| 6 | 4 | Control | 1.42 |
| 7 | 1 | Tr1 | 3.33 |
| 7 | 2 | Control | 0 |
| 7 | 3 | Control | 0 |
| 7 | 4 | Control | 0 |
| 8 | 1 | Tr1 | 1.88 |
| 8 | 2 | Control | 0.03 |
| 8 | 3 | Control | 1.03 |
| 8 | 4 | Control | 1 |
| 9 | 1 | Tr1 | 8.5 |
| 9 | 2 | Control | 0 |
| 9 | 3 | Control | 0.37 |
| 9 | 4 | Control | 0 |
| 10 | 1 | Tr1 | 2.27 |
| 10 | 2 | Control | 0 |
| 10 | 3 | Control | 0 |
| 10 | 4 | Control | 0 |

The raw means are:

Fig. B

Analysing the data on the square root scale there was an interaction (p < 0.001, F-test) between the female population and the type of male synthetic pheromone blend with the main effects of male blend also being significant (p < 0.001, F-test) but the female population being only marginal (p = 0.095, F-test). The predicted means for comparisons are:

Fig. C

LSD1 (5%) = 0.4258 for comparison of treatments to controls, LSD2 (5%) = 0.5714 for comparison between treatments, LSD3 (5%) = 0.1906 for comparison between controls (329 df).

**Section 2. Pairwise assays**

Raw data (values=time in minute)

| **Bean females to bean (Tr1) vs chickpea (Tr2) blend** | | | |
| --- | --- | --- | --- |
| replicate | Area | Treatment | time spent |
| 1 | 1 | Tr1 | 0.97 |
| 1 | 2 | Control | 0 |
| 1 | 3 | Tr2 | 12.5 |
| 1 | 4 | Control | 0.3 |
| 2 | 1 | Tr1 | 4.4 |
| 2 | 2 | Control | 0.48 |
| 2 | 3 | Tr2 | 9.85 |
| 2 | 4 | Control | 0.75 |
| 3 | 1 | Tr1 | 7.42 |
| 3 | 2 | Control | 0.38 |
| 3 | 3 | Tr2 | 6.13 |
| 3 | 4 | Control | 0.22 |
| 4 | 1 | Tr1 | 1.22 |
| 4 | 2 | Control | 3.68 |
| 4 | 3 | Tr2 | 0 |
| 4 | 4 | Control | 0.33 |
| 5 | 1 | Tr1 | 5.53 |
| 5 | 2 | Control | 0 |
| 5 | 3 | Tr2 | 3.85 |
| 5 | 4 | Control | 2.7 |
| 6 | 1 | Tr1 | 10.65 |
| 6 | 2 | Control | 0.1 |
| 6 | 3 | Tr2 | 1.05 |
| 6 | 4 | Control | 0 |
| 7 | 1 | Tr1 | 2.63 |
| 7 | 2 | Control | 0 |
| 7 | 3 | Tr2 | 6.4 |
| 7 | 4 | Control | 0 |
| 8 | 1 | Tr1 | 4.52 |
| 8 | 2 | Control | 1.92 |
| 8 | 3 | Tr2 | 5.83 |
| 8 | 4 | Control | 1 |
| 9 | 1 | Tr1 | 5.28 |
| 9 | 2 | Control | 0 |
| 9 | 3 | Tr2 | 3.65 |
| 9 | 4 | Control | 0.02 |
| 10 | 1 | Tr1 | 2.48 |
| 10 | 2 | Control | 0.43 |
| 10 | 3 | Tr2 | 3.42 |
| 10 | 4 | Control | 2.92 |
| **Bean females to bean (Tr1) vs chickpea-bean (Tr2) blend** | | | |
| replicate | Area | Treatment | time spent |
| 1 | 1 | Tr1 | 2.52 |
| 1 | 2 | Control | 0.5 |
| 1 | 3 | Tr2 | 3.32 |
| 1 | 4 | Control | 0.95 |
| 2 | 1 | Tr1 | 1.85 |
| 2 | 2 | Control | 0.37 |
| 2 | 3 | Tr2 | 5.73 |
| 2 | 4 | Control | 1.95 |
| 3 | 1 | Tr1 | 3.12 |
| 3 | 2 | Control | 2.43 |
| 3 | 3 | Tr2 | 0.95 |
| 3 | 4 | Control | 2.75 |
| 4 | 1 | Tr1 | 4.12 |
| 4 | 2 | Control | 1.6 |
| 4 | 3 | Tr2 | 6.72 |
| 4 | 4 | Control | 2.66 |
| 5 | 1 | Tr1 | 4.02 |
| 5 | 2 | Control | 0.4 |
| 5 | 3 | Tr2 | 3.8 |
| 5 | 4 | Control | 0 |
| 6 | 1 | Tr1 | 3.53 |
| 6 | 2 | Control | 1.12 |
| 6 | 3 | Tr2 | 6.08 |
| 6 | 4 | Control | 0.77 |
| 7 | 1 | Tr1 | 5.98 |
| 7 | 2 | Control | 0.25 |
| 7 | 3 | Tr2 | 4.48 |
| 7 | 4 | Control | 0.73 |
| 8 | 1 | Tr1 | 10.9 |
| 8 | 2 | Control | 0 |
| 8 | 3 | Tr2 | 2.38 |
| 8 | 4 | Control | 0.35 |
| 9 | 1 | Tr1 | 2.95 |
| 9 | 2 | Control | 0 |
| 9 | 3 | Tr2 | 4.67 |
| 9 | 4 | Control | 3.87 |
| 10 | 1 | Tr1 | 4.17 |
| 10 | 2 | Control | 0.75 |
| 10 | 3 | Tr2 | 6.12 |
| 10 | 4 | Control | 2.32 |

| **Bean females to chickpea (Tr1) vs chickpea-bean (Tr2) blend** | | | |
| --- | --- | --- | --- |
| replicate | Area | Treatment | time spent |
| 1 | 1 | Tr1 | 3.03 |
| 1 | 2 | Control | 1.6 |
| 1 | 3 | Tr2 | 2.8 |
| 1 | 4 | Control | 2.52 |
| 2 | 1 | Tr1 | 10 |
| 2 | 2 | Control | 2.2 |
| 2 | 3 | Tr2 | 1.4 |
| 2 | 4 | Control | 0.9 |
| 3 | 1 | Tr1 | 2.12 |
| 3 | 2 | Control | 1.1 |
| 3 | 3 | Tr2 | 5.4 |
| 3 | 4 | Control | 1.55 |
| 4 | 1 | Tr1 | 0 |
| 4 | 2 | Control | 1 |
| 4 | 3 | Tr2 | 1.53 |
| 4 | 4 | Control | 0 |
| 5 | 1 | Tr1 | 5.88 |
| 5 | 2 | Control | 0.07 |
| 5 | 3 | Tr2 | 2.65 |
| 5 | 4 | Control | 0.6 |
| 6 | 1 | Tr1 | 0 |
| 6 | 2 | Control | 0.05 |
| 6 | 3 | Tr2 | 11.93 |
| 6 | 4 | Control | 0.05 |
| 7 | 1 | Tr1 | 7.07 |
| 7 | 2 | Control | 1.25 |
| 7 | 3 | Tr2 | 0.23 |
| 7 | 4 | Control | 0 |
| 8 | 1 | Tr1 | 4.96 |
| 8 | 2 | Control | 2.07 |
| 8 | 3 | Tr2 | 4.7 |
| 8 | 4 | Control | 0 |
| 9 | 1 | Tr1 | 2.62 |
| 9 | 2 | Control | 0.58 |
| 9 | 3 | Tr2 | 5.05 |
| 9 | 4 | Control | 0.17 |
| 10 | 1 | Tr1 | 6.05 |
| 10 | 2 | Control | 3.7 |
| 10 | 3 | Tr2 | 1.43 |
| 10 | 4 | Control | 1.15 |

| **Chickpea females to bean (Tr1) vs chickpea (Tr2) blend** | | | |
| --- | --- | --- | --- |
| replicate | Area | Treatment | time spent |
| 1 | 1 | Tr1 | 2 |
| 1 | 2 | Control | 0.73 |
| 1 | 3 | Tr2 | 7.07 |
| 1 | 4 | Control | 1.18 |
| 2 | 1 | Tr1 | 4.17 |
| 2 | 2 | Control | 0.72 |
| 2 | 3 | Tr2 | 3.3 |
| 2 | 4 | Control | 0.58 |
| 3 | 1 | Tr1 | 1.17 |
| 3 | 2 | Control | 0.25 |
| 3 | 3 | Tr2 | 9.48 |
| 3 | 4 | Control | 0 |
| 4 | 1 | Tr1 | 4.42 |
| 4 | 2 | Control | 0.5 |
| 4 | 3 | Tr2 | 2.87 |
| 4 | 4 | Control | 1.75 |
| 5 | 1 | Tr1 | 2.2 |
| 5 | 2 | Control | 1.17 |
| 5 | 3 | Tr2 | 9.5 |
| 5 | 4 | Control | 0.57 |
| 6 | 1 | Tr1 | 1.92 |
| 6 | 2 | Control | 0.93 |
| 6 | 3 | Tr2 | 2.8 |
| 6 | 4 | Control | 6.5 |
| 7 | 1 | Tr1 | 0.02 |
| 7 | 2 | Control | 0 |
| 7 | 3 | Tr2 | 3.58 |
| 7 | 4 | Control | 1.4 |
| 8 | 1 | Tr1 | 6.68 |
| 8 | 2 | Control | 1.45 |
| 8 | 3 | Tr2 | 6.33 |
| 8 | 4 | Control | 1.48 |
| 9 | 1 | Tr1 | 2.48 |
| 9 | 2 | Control | 0.53 |
| 9 | 3 | Tr2 | 4.73 |
| 9 | 4 | Control | 0.3 |
| 10 | 1 | Tr1 | 0.95 |
| 10 | 2 | Control | 0.13 |
| 10 | 3 | Tr2 | 6.23 |
| 10 | 4 | Control | 2.4 |

| **Chickpea females to bean (Tr1) vs chickpea-bean (Tr2) blend** | | | |
| --- | --- | --- | --- |
| replicate | Area | Treatment | time spent |
| 1 | 1 | Tr1 | 3.48 |
| 1 | 2 | Control | 0.63 |
| 1 | 3 | Tr2 | 5.77 |
| 1 | 4 | Control | 1.02 |
| 2 | 1 | Tr1 | 10 |
| 2 | 2 | Control | 0 |
| 2 | 3 | Tr2 | 2.15 |
| 2 | 4 | Control | 0 |
| 3 | 1 | Tr1 | 4 |
| 3 | 2 | Control | 3 |
| 3 | 3 | Tr2 | 2.2 |
| 3 | 4 | Control | 0 |
| 4 | 1 | Tr1 | 1.76 |
| 4 | 2 | Control | 1.7 |
| 4 | 3 | Tr2 | 2.98 |
| 4 | 4 | Control | 1.6 |
| 5 | 1 | Tr1 | 3.68 |
| 5 | 2 | Control | 0.22 |
| 5 | 3 | Tr2 | 5.38 |
| 5 | 4 | Control | 0.42 |
| 6 | 1 | Tr1 | 0 |
| 6 | 2 | Control | 0 |
| 6 | 3 | Tr2 | 11 |
| 6 | 4 | Control | 0.35 |
| 7 | 1 | Tr1 | 0 |
| 7 | 2 | Control | 0 |
| 7 | 3 | Tr2 | 10.1 |
| 7 | 4 | Control | 0 |
| 8 | 1 | Tr1 | 2.75 |
| 8 | 2 | Control | 1.7 |
| 8 | 3 | Tr2 | 4.13 |
| 8 | 4 | Control | 2.78 |
| 9 | 1 | Tr1 | 0 |
| 9 | 2 | Control | 0.88 |
| 9 | 3 | Tr2 | 6.03 |
| 9 | 4 | Control | 1.2 |
| 10 | 1 | Tr1 | 0 |
| 10 | 2 | Control | 0 |
| 10 | 3 | Tr2 | 1.37 |
| 10 | 4 | Control | 0 |

| **Chickpea females to chickpea (Tr1) vs chickpea-bean (Tr2) blend** | | | |
| --- | --- | --- | --- |
| replicate | Area | Treatment | time spent |
| 1 | 1 | Tr1 | 5.45 |
| 1 | 2 | Control | 3.75 |
| 1 | 3 | Tr2 | 4.43 |
| 1 | 4 | Control | 1.37 |
| 2 | 1 | Tr1 | 6.87 |
| 2 | 2 | Control | 0.33 |
| 2 | 3 | Tr2 | 6.57 |
| 2 | 4 | Control | 0 |
| 3 | 1 | Tr1 | 4.49 |
| 3 | 2 | Control | 0.9 |
| 3 | 3 | Tr2 | 4.82 |
| 3 | 4 | Control | 2.87 |
| 4 | 1 | Tr1 | 4.27 |
| 4 | 2 | Control | 2.3 |
| 4 | 3 | Tr2 | 3.38 |
| 4 | 4 | Control | 0.58 |
| 5 | 1 | Tr1 | 5.18 |
| 5 | 2 | Control | 0.07 |
| 5 | 3 | Tr2 | 5.53 |
| 5 | 4 | Control | 0.75 |
| 6 | 1 | Tr1 | 3.3 |
| 6 | 2 | Control | 1.52 |
| 6 | 3 | Tr2 | 1.2 |
| 6 | 4 | Control | 2.39 |
| 7 | 1 | Tr1 | 3.33 |
| 7 | 2 | Control | 3.4 |
| 7 | 3 | Tr2 | 7.25 |
| 7 | 4 | Control | 1.1 |
| 8 | 1 | Tr1 | 6.68 |
| 8 | 2 | Control | 1.45 |
| 8 | 3 | Tr2 | 6.33 |
| 8 | 4 | Control | 1.48 |
| 9 | 1 | Tr1 | 2.48 |
| 9 | 2 | Control | 0.53 |
| 9 | 3 | Tr2 | 2.73 |
| 9 | 4 | Control | 0.3 |
| 10 | 1 | Tr1 | 6.3 |
| 10 | 2 | Control | 0.13 |
| 10 | 3 | Tr2 | 6.23 |
| 10 | 4 | Control | 2.4 |

| **Chickpea-bean females to bean (Tr1) vs chickpea (Tr2) blend** | | | |
| --- | --- | --- | --- |
| replicate | Area | Treatment | time spent |
| 1 | 1 | Tr1 | 0.95 |
| 1 | 2 | Control | 0 |
| 1 | 3 | Tr2 | 9.47 |
| 1 | 4 | Control | 0 |
| 2 | 1 | Tr1 | 0.95 |
| 2 | 2 | Control | 0 |
| 2 | 3 | Tr2 | 5.45 |
| 2 | 4 | Control | 0 |
| 3 | 1 | Tr1 | 0 |
| 3 | 2 | Control | 0.58 |
| 3 | 3 | Tr2 | 3.81 |
| 3 | 4 | Control | 0.53 |
| 4 | 1 | Tr1 | 2.27 |
| 4 | 2 | Control | 0.7 |
| 4 | 3 | Tr2 | 0.92 |
| 4 | 4 | Control | 0.33 |
| 5 | 1 | Tr1 | 2.73 |
| 5 | 2 | Control | 2.03 |
| 5 | 3 | Tr2 | 2.05 |
| 5 | 4 | Control | 1.6 |
| 6 | 1 | Tr1 | 0.52 |
| 6 | 2 | Control | 1 |
| 6 | 3 | Tr2 | 5.73 |
| 6 | 4 | Control | 0.75 |
| 7 | 1 | Tr1 | 5.35 |
| 7 | 2 | Control | 0.42 |
| 7 | 3 | Tr2 | 3.12 |
| 7 | 4 | Control | 1.27 |
| 8 | 1 | Tr1 | 0 |
| 8 | 2 | Control | 0 |
| 8 | 3 | Tr2 | 3.63 |
| 8 | 4 | Control | 0 |
| 9 | 1 | Tr1 | 0.97 |
| 9 | 2 | Control | 0.27 |
| 9 | 3 | Tr2 | 9.05 |
| 9 | 4 | Control | 0.22 |
| 10 | 1 | Tr1 | 0.35 |
| 10 | 2 | Control | 0 |
| 10 | 3 | Tr2 | 5.82 |
| 10 | 4 | Control | 0.63 |

| **Chickpea-bean females to bean (Tr1) vs chickpea-bean (Tr2) blend** | | | |
| --- | --- | --- | --- |
| replicate | Area | Treatment | time spent |
| 1 | 1 | Tr1 | 0 |
| 1 | 2 | Control | 0 |
| 1 | 3 | Tr2 | 3.23 |
| 1 | 4 | Control | 0 |
| 2 | 1 | Tr1 | 2.3 |
| 2 | 2 | Control | 0 |
| 2 | 3 | Tr2 | 4.72 |
| 2 | 4 | Control | 1.18 |
| 3 | 1 | Tr1 | 4 |
| 3 | 2 | Control | 0 |
| 3 | 3 | Tr2 | 2.7 |
| 3 | 4 | Control | 2 |
| 4 | 1 | Tr1 | 0 |
| 4 | 2 | Control | 0 |
| 4 | 3 | Tr2 | 2.45 |
| 4 | 4 | Control | 1.2 |
| 5 | 1 | Tr1 | 1.67 |
| 5 | 2 | Control | 0.93 |
| 5 | 3 | Tr2 | 0.92 |
| 5 | 4 | Control | 0.43 |
| 6 | 1 | Tr1 | 0 |
| 6 | 2 | Control | 0.02 |
| 6 | 3 | Tr2 | 8.8 |
| 6 | 4 | Control | 0.47 |
| 7 | 1 | Tr1 | 0 |
| 7 | 2 | Control | 0 |
| 7 | 3 | Tr2 | 4.02 |
| 7 | 4 | Control | 0.25 |
| 8 | 1 | Tr1 | 1.28 |
| 8 | 2 | Control | 1.83 |
| 8 | 3 | Tr2 | 4.52 |
| 8 | 4 | Control | 0 |
| 9 | 1 | Tr1 | 5.88 |
| 9 | 2 | Control | 0 |
| 9 | 3 | Tr2 | 1.88 |
| 9 | 4 | Control | 0 |
| 10 | 1 | Tr1 | 2.55 |
| 10 | 2 | Control | 0.13 |
| 10 | 3 | Tr2 | 0.95 |
| 10 | 4 | Control | 0.88 |

| **Chickpea-bean females to chickpea (Tr1) vs chickpea-bean (Tr2) blend** | | | |
| --- | --- | --- | --- |
| replicate | Area | Treatment | time spent |
| 1 | 1 | Tr1 | 0 |
| 1 | 2 | Control | 0.07 |
| 1 | 3 | Tr2 | 0 |
| 1 | 4 | Control | 0.77 |
| 2 | 1 | Tr1 | 2.53 |
| 2 | 2 | Control | 0.78 |
| 2 | 3 | Tr2 | 9 |
| 2 | 4 | Control | 0.63 |
| 3 | 1 | Tr1 | 0.88 |
| 3 | 2 | Control | 1.05 |
| 3 | 3 | Tr2 | 6.23 |
| 3 | 4 | Control | 1.63 |
| 4 | 1 | Tr1 | 0 |
| 4 | 2 | Control | 0 |
| 4 | 3 | Tr2 | 13.25 |
| 4 | 4 | Control | 0 |
| 5 | 1 | Tr1 | 3.2 |
| 5 | 2 | Control | 0 |
| 5 | 3 | Tr2 | 1.28 |
| 5 | 4 | Control | 0 |
| 6 | 1 | Tr1 | 3.02 |
| 6 | 2 | Control | 1.72 |
| 6 | 3 | Tr2 | 2.72 |
| 6 | 4 | Control | 0.88 |
| 7 | 1 | Tr1 | 2.42 |
| 7 | 2 | Control | 1.2 |
| 7 | 3 | Tr2 | 2.17 |
| 7 | 4 | Control | 0.33 |
| 8 | 1 | Tr1 | 12.1 |
| 8 | 2 | Control | 0 |
| 8 | 3 | Tr2 | 0.2 |
| 8 | 4 | Control | 0.4 |
| 9 | 1 | Tr1 | 4.73 |
| 9 | 2 | Control | 1.58 |
| 9 | 3 | Tr2 | 3.03 |
| 9 | 4 | Control | 0.45 |
| 10 | 1 | Tr1 | 0 |
| 10 | 2 | Control | 0 |
| 10 | 3 | Tr2 | 11 |
| 10 | 4 | Control | 0 |

The raw means and SEs are:

Fig. D

Analysing the data on the square root scale there was an interaction (p = 0.009, F-test) between the female population and the type of male synthetic pheromone blend with both main effects also being significant (p < 0.001, F-test, for the female population and p < 0.001, F-test, for the type of male synthetic blend). The predicted means for comparisons are:

Fig. E

LSD1 (5%) = 0.3573 for comparison of treatments to controls, LSD2 (5%) = 0.4288 for comparison between treatments, LSD3 (5%) = 0.2414 for comparison between controls (309 df).

**Section 3. All Data**

Using all the data from the assays, the raw data means and SEs are:

Fig. F

Fig. G

LSD1 (5%) = 0.2778 for comparison of treatments to controls, LSD2 (5%) = 0.3582 for comparison between treatments, LSD3 (5%) = 0.1581 for comparison between controls (663 df).

**Section 4. Generation Effect**

Raw data (values=time in minute)

| **Chickpea females to bean (Tr1) vs chickpea (Tr2) blend** | | | |
| --- | --- | --- | --- |
| replicate | Area | Treatment | time spent |
| 1 | 1 | Tr1 | 11.12 |
| 1 | 2 | Control | 0.56 |
| 1 | 3 | Tr2 | 0 |
| 1 | 4 | Control | 0.85 |
| 2 | 1 | Tr1 | 0.12 |
| 2 | 2 | Control | 0.22 |
| 2 | 3 | Tr2 | 5.97 |
| 2 | 4 | Control | 3.27 |
| 3 | 1 | Tr1 | 5.37 |
| 3 | 2 | Control | 0.72 |
| 3 | 3 | Tr2 | 4.85 |
| 3 | 4 | Control | 0 |
| 4 | 1 | Tr1 | 0 |
| 4 | 2 | Control | 3.18 |
| 4 | 3 | Tr2 | 5.4 |
| 4 | 4 | Control | 0 |
| 5 | 1 | Tr1 | 0.37 |
| 5 | 2 | Control | 1.6 |
| 5 | 3 | Tr2 | 3.45 |
| 5 | 4 | Control | 3.52 |
| 6 | 1 | Tr1 | 0 |
| 6 | 2 | Control | 1.85 |
| 6 | 3 | Tr2 | 8.92 |
| 6 | 4 | Control | 0.73 |
| 7 | 1 | Tr1 | 1.18 |
| 7 | 2 | Control | 0.12 |
| 7 | 3 | Tr2 | 8.43 |
| 7 | 4 | Control | 0.87 |
| 8 | 1 | Tr1 | 2.4 |
| 8 | 2 | Control | 0 |
| 8 | 3 | Tr2 | 4.2 |
| 8 | 4 | Control | 0.74 |
| 9 | 1 | Tr1 | 2.07 |
| 9 | 2 | Control | 0.18 |
| 9 | 3 | Tr2 | 7.02 |
| 9 | 4 | Control | 4.7 |
| 10 | 1 | Tr1 | 3.2 |
| 10 | 2 | Control | 0.65 |
| 10 | 3 | Tr2 | 4.03 |
| 10 | 4 | Control | 1.63 |

The raw means for the data are:

Fig. H

Analysing the time spent data, there was only a main effect of blend (p < 0.001, F-test) and no effect of the number of generations (p = 0.439, F-test) or an interaction between the two factors (p = 0.799, F-test). The means on the square root scale are:

Fig. I

LSD1 (5%) for comparison of treatments to control = 0.4207, LSD2 (5%) for comparison of treatments = 0.4858 (56 df).
