# Supplementary material for: Host shift induces changes in mate choice of the seed predator Acanthoscelides obtectus via altered chemical signalling
Source: PLoS One. 2018 Nov 14;13(11):e0206144. doi: 10.1371/journal.pone.0206144 (PMC6235263; doi:10.1371/journal.pone.0206144)
Supplement: S3 File — (DOCX) [file pone.0206144.s004.docx]

**Host shift induces changes in mate choice of the seed predator *Acanthoscelides obtectus* via altered chemical signalling**

József Vuts, Christine M. Woodcock, Lisa König, Stephen J. Powers, John A. Pickett, Árpád Szentesi, Michael A. Birkett

**Still-air bioassays**


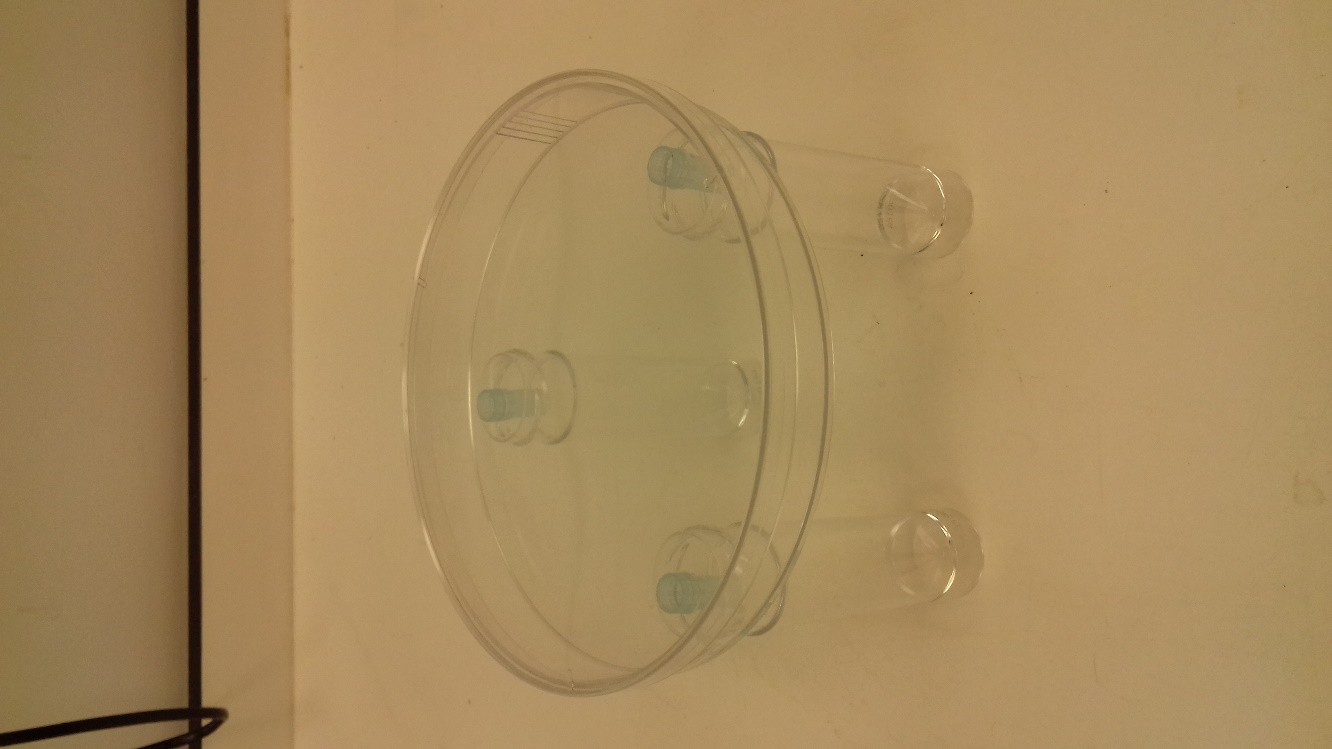


Fig. A

Raw data (values=number of individuals)

|  |  | female host line | |
| --- | --- | --- | --- |
| replicate | treatment | bean | chickpea |
| 1 | control | 2 | 0 |
| 1 | bean blend | 2 | 2 |
| 1 | chickpea blend | 6 | 8 |
| 1 | non-responders | 0 | 0 |
| 2 | control | 1 | 1 |
| 2 | bean blend | 5 | 3 |
| 2 | chickpea blend | 4 | 3 |
| 2 | non-responders | 0 | 3 |
| 3 | control | 2 | 3 |
| 3 | bean blend | 2 | 1 |
| 3 | chickpea blend | 4 | 4 |
| 3 | non-responders | 2 | 2 |
| 4 | control | 1 | 0 |
| 4 | bean blend | 5 | 4 |
| 4 | chickpea blend | 4 | 5 |
| 4 | non-responders | 0 | 1 |
| 5 | control | 1 | 1 |
| 5 | bean blend | 5 | 2 |
| 5 | chickpea blend | 4 | 7 |
| 5 | non-responders | 0 | 0 |
| 6 | control | 3 | 3 |
| 6 | bean blend | 3 | 1 |
| 6 | chickpea blend | 4 | 6 |
| 6 | non-responders | 0 | 0 |
| 7 | control | 3 | 0 |
| 7 | bean blend | 3 | 4 |
| 7 | chickpea blend | 3 | 6 |
| 7 | non-responders | 1 | 0 |

The Genstat (18^th^ edition, © VSN International Ltd, Hemel Hempstead, UK) statistical package was used for the analysis.

Firstly, we can note that totalling over the seven replicates that each use 20 beetles, 10 beetles of each population, the values are:

| **Treatment (Blend)** | **Female** |  |  |
| --- | --- | --- | --- |
|  | **Bean** | **Chickpea** | **Totals** |
| Control | 13 | 8 | **21** |
| Bean blend | 25 | 17 | **42** |
| Chickpea blend | 29 | 39 | **68** |
| NonResp. (Petri Dish) | 3 | 6 | **9** |
| **Total** | **70** | **70** | **140** |

Having fitted the generalized linear mixed model, which effectively averages over the seven replicates rather than summing over them, there was some partial evidence of an interaction between treatment (blend) and female population (p = 0.156, Chi-squared test). The predicted means are:

**Female Bean Chickpea**

**Prediction s.e. Prediction s.e.**

**Treatment**

**Bean blend** **1.2730** 0.2000 **0.8873** 0.2425

**Chickpea blend** **1.4214** 0.1857 **1.7177** 0.1601

**Control** 0.6190 0.2773 0.1335 0.3533

**NonResp.(Petri Dish)** -0.8473 0.5761 -0.1541 0.4078

These means can be compared using the appropriate least significant difference (LSD) value given in the matrix below, reading along the appropriate row and up the appropriate column. The Chickpea females preferred the chickpea blend, with significance difference (p < 0.05, LSD) over the bean blend. There was no significant difference (p > 0.05 LSD) between bean and chickpea blends for the Bean female population.

Least significant differences of predictions (5% level)

-------------------------------------------------------

Treatment bean blend Female Bean 1 *

Treatment bean blend Female Chickpea 2 0.6344 *

Treatment chickpea blend Female Bean 3 **0.5508** 0.6164

Treatment chickpea blend Female Chickpea 4 0.5170 **0.5865**

Treatment control Female Bean 5 0.6901 0.7435

Treatment control Female Chickpea 6 0.8194 0.8649

Treatment NonResp(PetriDish) Female Bean 7 1.2306 1.2614

Treatment NonResp(PetriDish) Female Chickpea 8 0.9166 0.9575

1 2

Treatment chickpea blend Female Bean 3 *

Treatment chickpea blend Female Chickpea 4 0.4948 *

Treatment control Female Bean 5 0.6736 0.6463

Treatment control Female Chickpea 6 0.8055 0.7829

Treatment NonResp(PetriDish) Female Bean 7 1.2215 1.2067

Treatment NonResp(PetriDish) Female Chickpea 8 0.9042 0.8841

3 4

Treatment control Female Bean 5 *

Treatment control Female Chickpea 6 0.9065 *

Treatment NonResp(PetriDish) Female Bean 7 1.2903 1.3638

Treatment NonResp(PetriDish) Female Chickpea 8 0.9952 1.0889

5 6

Treatment NonResp(PetriDish) Female Bean 7 *

Treatment NonResp(PetriDish) Female Chickpea 8 1.4244 *

7 8

The back-transformed means for presentation are:

**Female Bean Chickpea**

**Prediction s.e. Prediction s.e.**

**Treatment**

**Bean blend** 3.571 0.7143 2.429 0.5890

**Chickpea blend** 4.143 0.7693 5.571 0.8921

**Control** 1.857 0.5151 1.143 0.4038

**NonResp.(Petri Dish)** 0.429 0.2469 0.857 0.3495

Turning this into a picture gives:

Fig. B
