# Supplementary material for: Host shift induces changes in mate choice of the seed predator Acanthoscelides obtectus via altered chemical signalling
Source: PLoS One. 2018 Nov 14;13(11):e0206144. doi: 10.1371/journal.pone.0206144 (PMC6235263; doi:10.1371/journal.pone.0206144)
Supplement: S4 File — (DOCX) [file pone.0206144.s005.docx]

**Host shift induces changes in mate choice of the seed predator *Acanthoscelides obtectus* via altered chemical signalling**

József Vuts, Christine M. Woodcock, Lisa König, Stephen J. Powers, John A. Pickett, Árpád Szentesi, Michael A. Birkett

Raw data (values=amplitudes in mV of EAG responses)

| replicate | stimulus | EAG response amplitude (mV) | | |
| --- | --- | --- | --- | --- |
|  |  | bean females | chickpea females | chickpea/bean females |
| 1 | air (control) | 1.06 | 1.281 | 1.415 |
| 1 | bean males | 1.116 | 1.413 | 1.802 |
| 1 | chickpea males | 1.066 | 1.237 | 1.791 |
| 1 | chickpea/bean males | 1.083 | 1.286 | 1.433 |
| 2 | air (control) | 0.959 | 1.558 | 0.995 |
| 2 | bean males | 1.339 | 1.943 | 1.381 |
| 2 | chickpea males | 1.022 | 1.757 | 1.299 |
| 2 | chickpea/bean males | 1.203 | 1.679 | 1.3 |
| 3 | air (control) | 1.334 | 1.192 | 1.061 |
| 3 | bean males | 1.593 | 1.411 | 1.152 |
| 3 | chickpea males | 1.299 | 1.266 | 1.111 |
| 3 | chickpea/bean males | 1.429 | 1.192 | 1.086 |
| 4 | air (control) | 1.189 | 1.271 | 1.19 |
| 4 | bean males | 1.275 | 2.093 | 2.068 |
| 4 | chickpea males | 1.291 | 1.697 | 2.062 |
| 4 | chickpea/bean males | 1.238 | 1.497 | 1.893 |
| 5 | air (control) | 1.497 | 1.272 | 1.576 |
| 5 | bean males | 1.951 | 1.404 | 2.139 |
| 5 | chickpea males | 1.466 | 1.325 | 1.867 |
| 5 | chickpea/bean males | 1.401 | 1.332 | 1.643 |
